# Area-level socioeconomic characteristics and incidence of metabolic syndrome: a prospective cohort study

**DOI:** 10.1186/1471-2458-13-681

**Published:** 2013-07-25

**Authors:** Anh D Ngo, Catherine Paquet, Natasha J Howard, Neil T Coffee, Robert Adams, Anne Taylor, Mark Daniel

**Affiliations:** 1 Social Epidemiology and Evaluation Research Group, Sansom Institute for Health Research, and School of Population Health, University of South Australia, Adelaide 5001, Australia; 2 Research Centre of the Douglas Mental Health University Institute, Verdun, Québec H4H 1R2, Canada; 3 The Health Observatory, Discipline of Medicine, The University of Adelaide, Adelaide, South Australia 5005, Australia; 4 Population Research and Outcome Studies, Discipline of Medicine, The University of Adelaide, Adelaide, South Australia 5005, Australia; 5 Department of Medicine, The University of Melbourne, St Vincent’s Hospital, Melbourne, Victoria 3065, Australia; 6 Social Epidemiology and Evaluation Research Group, Sansom Institute for Health Research, and School of Population Health, University of South Australia, P4-18F, Playford Building, City East Campus, Adelaide 5000, Australia

**Keywords:** Metabolic syndrome, Incidence, Socioeconomic status, Income, Education, Cohort study, Residence characteristics

## Abstract

**Background:**

The evidence linking socioeconomic environments and metabolic syndrome (MetS) has primarily been based on cross-sectional studies. This study prospectively examined the relationships between area-level socioeconomic position (SEP) and the incidence of MetS.

**Methods:**

A prospective cohort study design was employed involving 1,877 men and women aged 18+ living in metropolitan Adelaide, Australia, all free of MetS at baseline. Area-level SEP measures, derived from Census data, included proportion of residents completing a university education, and median household weekly income. MetS, defined according to International Diabetes Federation, was ascertained after an average of 3.6 years follow up. Associations between each area-level SEP measure and incident MetS were examined by Poisson regression Generalised Estimating Equations models. Interaction between area- and individual-level SEP variables was also tested.

**Results:**

A total of 156 men (18.7%) and 153 women (13.1%) developed MetS. Each percentage increase in the proportion of residents with a university education corresponded to a 2% lower risk of developing MetS (age and sex-adjusted incidence risk ratio (RR) = 0.98; 95% confidence interval (CI) =0.97-0.99). This association persisted after adjustment for individual-level income, education, and health behaviours. There was no significant association between area-level income and incident MetS overall. For the high income participants, however, a one standard deviation increase in median household weekly income was associated with a 29% higher risk of developing MetS (Adjusted RR = 1.29; 95%CI = 1.04-1.60).

**Conclusions:**

While area-level education was independently and inversely associated with the risk of developing MetS, the association between area-level income and the MetS incidence was modified by individual-level income.

## Background

A growing literature has documented the relationship between individual and residential area (often referred to as “neighbourhood”) socioeconomic position (SEP) with a wide range of health outcomes, including cardiometabolic diseases (i.e., cardiovascular diseases and type 2 diabetes). Low neighbourhood SEP, for example, has been found to be associated with an increased incidence of ischaemic stroke [1], incidence of coronary heart disease (CHD) [2], and incidence of type 2 diabetes [3,4]. Although the underlying mechanisms involved in this relationship are not entirely elucidated [5-7], a growing body of evidence demonstrates that socioeconomic factors influence cardiometabolic health outcomes through shaping health related behaviours and psychological antecedents, and subsequently predicting biological risk factors [8-10]. Available research also indicates that area-level socioeconomic conditions can operate independently or interactively with individual-level SEP to determine whether a predisposition to developing cardiometabolic diseases is realised [11,12].

Metabolic syndrome (MetS) - a clustering of disturbed glucose and insulin metabolism, obesity or abdominal adiposity, dyslipidaemia, and hypertension, is an important risk factor for cardiometabolic diseases. There is relatively well-established evidence showing an inverse association between individual-level SEP and prevalence or incidence of MetS [13-26] or its components [8,27-35]. Also, a substantial body of literature has demonstrated that living in socioeconomically disadvantaged areas is associated with an increased prevalence or incidence of MetS components: prevalence of type 2 diabetes or insulin resistance [4,36-38], high blood pressure [36,38-43], and larger waist [41,44], and incidence of type 2 diabetes [3,4]. Some studies have also reported evidence of interaction between area- and individual-level SEP characteristics with associations either stronger or only present in low SEP individuals [12]. For example, associations between state-level income inequality and cardiometabolic risk factors (e.g., body mass index (BMI), hypertension, and sedentarism) have been found to be stronger in low income individuals [43]. However, the role of area-level SEP in shaping the distribution and development of MetS - a stronger risk marker of cardiometabolic disease has been infrequently examined. A recent review reported that out of 56 studies evaluating the influence of the socioeconomic environment on cardiometabolic risk factors, only two considered MetS as the outcome in analysis [12].

Four cross-sectional studies have evaluated the relationships between area-level socioeconomic conditions on prevalent MetS (or a cluster of the MetS components). The results of these studies have consistently shown an inverse association [45-48], but in one study [46], the strength of associations varied according to individual-level income and education. Being cross-sectional, these studies could not assess the predictive capacity of area-level SEP in relation to the development of MetS over time. Furthermore, available studies used a composite index or score to rate area-level SEP. While this method allowed for the evaluation of multiple features of area-level SES, it did not allow for the disentanglement of independent effects pertaining to specific features of area-level SEP on incident MetS. Thus, there is a need to conduct a longitudinal study to evaluate relationships between specific characteristics of area-level SEP and the development of MetS.

Characteristics of individual- and area-level SEP are interrelated. Individual SEP may influence the area where one chooses to reside, while area-level socioeconomic conditions both reflect and shape social and built environments of the residential area, and potentially affect the socioeconomic attainment of residents via educational and occupational opportunities [49]. Given these inter-relationships, attention is increasingly shifting to disentangling the independent and/or joint effect of individual SEP and residential area socioeconomic conditions on cardiometabolic outcomes [12,43,45]. To our knowledge, however, no studies have empirically tested whether area-level and individual-level SEP factors independently or interactively influence the development of MetS.

The purpose of this study was to go beyond individual cardiometabolic risks in examining the development of MetS as a cluster of risks and a stronger predictor of cardiometabolic disease in relation to area-level SEP factors. It sought to expand the literature by examining the influence of area-level SEP on incident MetS and exploring if this influence is independent of or differed by individual-level SEP. The aims of the study were: (i) to estimate the incidence of MetS in a population-based cohort; (ii) to examine the relationships between specific features of area-level SEP and incident MetS; and (iii) to determine whether these relationships were modified by individual-level measures of SEP. Hypotheses were: (i) area-level SEP characteristics would be inversely associated with the development of MetS; (ii) these associations would be either stronger or present only in low SEP individuals.

## Methods

### Study population

This study was conducted through the Place and Metabolic Syndrome (PAMS) project, a research initiative that aims to evaluate the relationships between local-area social and built environmental factors and cardiometabolic health. The project draws on the North West Adelaide Health Study (NWAHS), a population-based prospective cohort study designed to provide longitudinal self-reported and clinically measured data on a number of chronic health outcomes and disease risk factors [50]. Data collection was based in the northern and western regions of metropolitan Adelaide, the capital city of South Australia (SA). The study region accounts for 38% of the Adelaide metropolitan population and 28% of the state population [51]. Furthermore, the north-west region of Adelaide has been identified as having high proportions of low income families and the lowest full-time secondary school participation rates [52]. The eligible population included persons from households that, at study inception and time of baseline sampling, had a telephone with the corresponding number listed in the Electronic White Pages directory.

Baseline recruitment was carried out between 2000 and 2003. Telephone numbers of households in the study region listed in the Electronic White Pages directory were randomly selected. An invitation letter was sent to selected households followed within 10 days by a telephone call from trained health study recruiters. Initially, 8,213 residents aged 18 years or older from selected households were asked to participate in the study of whom 5,850 (71.2%) completed a computer-assisted telephone interview (CATI) (Additional file 1). Interview respondents were sent a questionnaire for self-reporting a number of chronic conditions and health risk factors (Additional file 2). Finally, consenting participants attended a biomedical examination that involved anthropometric measurements and blood draws to assay biochemical measures and determine cardiometabolic risk factors as well as MetS. At Wave 1, 4,056 participants (49.4% of the eligible sample, and 69.4% of those who completed the CATI) completed the examination.

Two additional waves of data collection were undertaken; Wave 2 (2004-06) and Wave 3 (2008-10). One hundred participants died and 22 withdrew from the study between the first and second waves of data collection, leaving 3,943 participants eligible for Wave 2 participation (Additional file 3). Of these participants, 3,206 (81%) attended a follow-up biomedical assessment. At each wave of data collection, based on residential address information, valid records were assigned a georeference to represent the participant’s longitude and latitude. The population for the present study comprised of baseline-MetS free participants who completed biomedical assessments at Wave 1 and 2. Ethics approval was obtained from three Human Research Ethics Committees: the University of South Australia, Central Northern Adelaide Health Service and the South Australian Department of Health and Aging. Written informed consent was obtained from all participants prior to data collection.

### Metabolic syndrome

MetS was defined, using the International Diabetes Foundation criteria [53], as abdominal obesity (waist circumference ≥94 cm for Europid men and ≥90 cm for non-Europid men, and ≥80 cm for Europid and non-Europid women) plus any two or more of the following four metabolic abnormalities: Hypertriglyceridemia (≥ 1.7 mmol/l) or specific treatment for this lipid abnormality; low high-density lipoprotein (HDL) cholesterol level (≤ 1.3 mmol in women, ≤ 1.0 mmol in men) or specific treatment for this lipid abnormality; elevated blood pressure (≥130/85 mm Hg) or treatment of previously diagnosed hypertension; and hyperglycaemia (fasting plasma glucose > 6.1 mmol/l) or previously diagnosed type 2 diabetes.

### Area-level socioeconomic position

For this research, the spatial unit was the 2001 State Suburb (SSC), an Australian Bureau of Statistics (ABS) Census Geographic Unit [54] which approximates to administrative boundaries (i.e., suburbs) within urban areas. The use of State Suburbs to express environmental scale provides adequate numbers of participants within area-level units, in addition to reasonable between unit variability. The study region consisted of 154 State Suburbs, with a median population of 2,027 persons. A geographic information system (GIS) was utilised to assign each participant to a State Suburb based on the Wave 1 georeference. Area-level SEP data represented at the State Suburb were obtained from the 2001 ABS Population and Housing Census. Two measures were extracted for analysis: the proportion of residents 18 years or older who had completed a university degree, and median household weekly income (in Australian Dollars (AUD)).

### Covariates

Covariates included in the analysis were measured at Wave 1. Individual-level SEP was represented in terms of self-reported education attainment (having a least a bachelor degree, or not) and gross annual household income classified as low (< 20,000 AUD); middle (20,000- 60,000 AUD); and high (> 60,000 AUD). These variables were also tested as potential effect modifiers of area-level SEP measures. Other individual covariates included age (in years), gender (male vs. female), and cardiometabolic risk behaviours. Smoking status was classified as current, former, and non- smokers. Alcohol consumption was defined as at risk and not at risk drinkers according to national guidelines [55]. Male participants were considered “at risk” if they consumed more than six standard drinks on any one day, an average of more than four drinks per day, or more than 28 standard drinks over a week. Female participants were coded “at risk” if they consumed more than four standard drinks on any one day, an average of more than two drinks per day, or more than 14 standard drinks over a week.

Health behaviours were ascertained from self-reported questions obtained from the ABS, National Health Survey Questionnaire and Guidelines [56]. The physical activity level was evaluated according to Metabolic Equivalence Tasks (METs) in hours per week [57] derived from the total amount and intensity of physical activity (walking, moderate and vigorous physical activity) carried out for sport, recreation or fitness within the last two weeks. Participants were assigned to either the sedentary, low, moderate, or high category if they achieved ≤100 METs, 100 - ≤1600 METs, 1600 - ≤3,200 METs, and >3,200 METs, respectively (Additional file 4).

### Statistical analysis

All analyses were performed with Stata version 11.0 [58]. Incident MetS was calculated by dividing the number of new cases by the number of individuals at risk. Descriptive statistics included frequency distributions for categorical variables (e.g., individual income and education), and determination of mean, median, and standard deviation for continuous variables (e.g., age, area-level income and education).

Associations between each area-level SEP characteristic and the incident of MetS were tested separately in four sequential statistical models. Poisson regression, conducted using Generalised Estimating Equation (GEE), was performed to compute incidence risk ratio (RR) and 95% Confidence Interval (CI). In model 1 (base model), we examined the association between the outcome variable and each area-level SEP variable, adjusted for age and gender. In model 2, we added individual SEP variables (i.e., income and education). In model 3 (full model), we added behavioural variables (i.e., smoking, alcohol consumption, and physical activity). In model 4, we added the other area-level SEP variable to test the relative importance of one component of area-level SEP over the other in predicting the incident MetS. Two-way interaction terms between area- and individual-level SEP variables were assessed for statistical significance. Analyses stratified by individual-level SEP were conducted when statistical evidence for an interactive effect (*p* < 0.05) was observed. To test if the association differed by gender, two-way interaction terms between area-level SEP characteristics and gender were also added to statistical models. GEE was used to account for the clustering of observations within spatial units (State Suburbs). This approach has been proposed to provide accurate estimates for the relationships between area-level characteristics and individual-level health outcomes in multilevel studies [59,60].

## Results

### Sample description

The sample available for the current analysis contained 2,586 participants, none of whom had MetS at baseline. At Wave 2, MetS status was determined for 1,991 participants (77%). Due to missing data for one or more of independent variables or covariates, a further 114 participants were removed from analysis. As a result, the final sample contained 1,877 participants residing in 143 State Suburbs. The median number of participants per spatial unit was 10, ranging from 1 to 56. The average follow up time was 3.6 years, varying from 1.8 to 5.9 years. Descriptive information on study participants and MetS components is presented in Table 1. Compared with participants who did not develop MetS, participants who developed MetS were older, reported a lower household income, and resided in areas with a lower income and a lower percentage of university graduates. Prevalence of MetS components at baseline was also higher in participants who developed MetS.

**Table 1:** Baseline characteristics of study participants by MetS status at Wave 2

**Characteristic**	**Wave 1 MetS free (n = 1877)**	**Not developing MetS (n = 1585)**	**Developing MetS (n = 292)**
	**Median (sd)/n (percent)**	**Median (sd)/n (percent)**	**Median (sd)/n (percent)**
**Age (years)**	47 (15.5)	45 (15.5)	55 (14.7)
**Gender**			
Male	816 (43.5)	663 (41.8)	153 (52.4)
Female	1,061 (56.5)	922 (58.2)	139 (47.6)
**Gross annual household income**			
<20,000 AUD	538 (28.7)	447 (28.2)	91 (31.2)
20,000-60,000 AUD	913 (48.6)	765 (43.8)	148 (50.7)
>60,000 AUD	426 (22.7)	373 (23.5)	53 (18.1)
**Individual education**			
Bachelor degree (Yes)	230 (12.2)	1,390 (87.7)	257 (88.0)
Bachelor degree (No)	1,647 (87.8)	195 (12.3)	35 (12.0)
**Physical Activity**			
Sedentary	524 (27.9)	440 (27.8)	84 (28.8)
Low exercise	669 (35.6)	577 (36.4)	92 (31.6)
Moderate exercise	515 (27.4)	435 (27.4)	80 (27.4)
High exercise	169 (9.0)	133 (8.4)	36 (12.3)
Smoking*			
Current	344 (18.3)	287 (18.1)	57 (19.5)
Former	669 (37.2)	661 (38.6)	88 (30.1)
Never	834 (44.4)	687 (43.3)	147 (50.3)
**Drinking**			
Not at risk	1,325 (70.6)	1,112 (70.2)	213 (73.0)
At risk	552 (29.4	473 (29.8)	79 (27.0)
**Area-level SEP**			
Percent university education or more	6.7 (4.8)	6.5 (4.7)	6.7 (4.8)
Median household weekly income (AUD)	671 (153)	673 (148)	660 (155)
**MetS component**			
Central obesity	884 (47.1)	671 (42.33)	213 (73.0)
Low HDL cholesterol	257 (12.7)	194 (12.2)	63 (21.58)
Hyperglycaemia	150 (8.0)	122 (7.7)	28 (9.6)
Hypertriglyceridemia	254 (13.5)	188 (11.9)	66 (22.6)
Hypertension	673 (35.9)	561 (34.2)	112 (47.66)

Regarding area-level SEP characteristics, the median of median household weekly income was 612 AUD (ranging from 361 to 1,323 AUD), and the median percentage of residents having a completed university education was 6.5% (ranging from 0 to 21.3%) (Table 1).

### Area-level SEP and MetS incidence

A total of 156 men (18.7%) and 153 women (13.1%) (15.6% of the study sample) developed MetS. As presented in Table 2, there was an inverse association between area-level education and the risk of MetS: each unit increase in the percentage of residents with a university qualification corresponded to a 2% lower risk of developing MetS (Model 1: RR = 0.98; 95%CI = 0.97-0.99). The statistical significance of this relationship was maintained following adjustments for individual-level SEP (Model 2), health risk behaviours (Model 3), and area-level income (Model 4). There was no statistical evidence (*p* ≥ 0.32) for an interaction between area-level education and either of the two individual-level SEP variables. Individual-level covariates beyond age and gender were not significantly associated with incident MetS.

**Table 2:** Association between area-level university education/income and MetS incidence (n = 1877)

	**Model 1**	**Model 2**	**Model 3**	**Model 4**
	**RR (95%CI)**	**RR (95%CI)**	**RR (95%CI)**	**RR (95%CI)**
**Area-level education**				
**Percent uni. education or more** (per 1% increase)	0.98 (0.97-0.99)^ a^	0.98 (0.96-0.99) ^a^	0.98 (0.96-0.99) ^a^	0.98 (0.96-0.99) ^a^
**Annual household income**				
<20,000 AUD	-	Ref	Ref	
20,000-60,000 AUD	-	1.04 (0.86-1.26)	1.00 (0.82-1.21)	1.00 (0.82-1.21)
>60,000 AUD	-	0.77 (0.55-1.08)	0.76 (0.54-1.08)	0.76 (0.54-1.07)
**Individual education**				
Bachelor degree (No)	-	Ref	Ref	
Bachelor degree (Yes)	-	1.28 (0.91-1.80)	1.23 (0.86-1.77)	1.24 (0.87-1.78)
**Area-level income**				
**Median household weekly income** (per 1 sd increase)	0.97 (0.88-1.07)	0.89 (0.75-1.07)	0.88 (0.74-1.05)	0.93 (0.78 -1.10)
**Annual household income**				
<20,000 AUD	-	Ref	Ref	
20,000-40,000 AUD	-	1.08 (0.88-1.32)	1.03 (0.85-1.26)	1.03 (0.84-1.25)
>60,000 AUD	-	0.69 (0.49-0.98) ^a^	0.68 (0.48-0.97) ^a^	0.69 (0.49-0.98) ^a^
Area-level income x Middle	-	1.04 (0.84-1.28)	1.04 (0.84-1.28)^ a^	1.02 (0.83-1.25)
Area-level x High	-	1.46 (1.11-1.91) ^b^	1.47 (1.12-1.92) ^b^	1.42 (1.01-1.85) ^b^
**Individual education**				
Bachelor degree (No)	-	Ref	Ref	Ref
Bachelor degree (Yes)	-	1.20 (0.86-1.66)	1.15 (0.81-1.64)	1.21 (0.85-1.74)

^a^*p* < 0.05; ^b^*p* < 0.01.

Model 1: Adjusted for age and gender.

Model 2: Adjusted for age and gender, and individual SEP.

Model 3: Adjusted for all variables in model 2 plus physical activity, smoking habit, and alcohol consumption.

Model 4: Adjusted for all variables in model 2 plus area-level income (for area-level education) or area-level education (for area-level income).

Table 3 summarises the results of statistical models assessing the effects of area-level income. All four models indicated no statistical evidence (*p* ≥ 0.15) for an association between area-level median household weekly income and the presence of MetS at the Wave 2 follow up. However, there was a statistically significant interaction (*p* = 0.005) between individual- and area-level income, but no evidence for an interaction between area-level income and individual-level education (*p* = 0.55). Among covariates other than age and gender, individual household income was significantly associated with incident MetS with the highest income participants having a 32% lower risk of developing MetS compared to those in the lowest income group (Full model: RR =0.68; 95% CI = 0.48-0.97). In all statistical models, there was no statistical evidence of an interaction between gender and any of area-level SEP measures (*p* ≥0.12).

**Table 3:** Association between area-level income and MetS incidence according to individual-level income

	**<20,00 AUD**	**20,000-60,00 AUD**	**>60,000 AUD**
	**RR (95%CI) (n = 538)**	**RR (95%CI) (n = 913)**	**RR (95%CI) (n = 426)**
**Model 1**			
**Median household weekly income** (per 1 sd increase)	0.90 (0.75-1.08)	0.92 (0.82-1.03)	1.31 (1.07-1.62) ^a^
**Model 2**			
**Median household weekly income** (per 1 sd increase)	0.87 (0.72-1.05)	0.92 (0.81-1.05)	1.31 (1.05-1.62) ^a^
**Individual-level education**			
Bachelor degree (No)			
Bachelor degree (Yes)	2.27 (1.08-4.80) ^a^	0.96 (1.57-1.62)	1.18 (0.71-1.96)
**Model 3**			
**Median household weekly income** (per 1 sd increase)	0.88 (0.73-1.05)	0.91 (0.80-1.04)	1.29 (1.04-1.60) ^a^
**Individual-level education**			
Bachelor degree (No)			
Bachelor degree (Yes)	2.54 (1.19-5.43) ^a^	0.94 (0.55-1.62)	0.94 (0.56-1.59)
**Model 4**			
**Median household weekly income** (per 1 sd increase)	0.92 (0.78 – 1.10)	0.98 (0.95 -1.00)	1.30 (1.05-1.62) ^a^
**Individual-level education**			
Bachelor degree (No)	Ref	Ref	Ref
Bachelor degree (Yes)	2.73 (1.23-6.06) ^a^	1.05 (0.61-1.81)	0.96 (0.57-1.61)

^a^*p* < 0.05.

Model 1: Adjusted for age and gender.

Model 2: Adjusted for age and gender, and individual SEP.

Model 3: Adjusted for all variables in model 2 plus physical activity, smoking habit, and alcohol consumption.

Model 4: Adjusted for all variables in model 2 plus area-level income (for area-level education) or area-level education (for area-level income).

To depict the nature of the interaction between area- and individual-level income, we conducted an analysis stratified according to individual household income (Table 3). A statistically significant association between area-level income and MetS incidence was observed for the high income participants. Specifically, a one standard deviation increase in area-level median household weekly income corresponded to a 31% higher risk of developing MetS (Model 1: RR = 1.31; 95%CI = 1.07-1.62) in this group. The association persisted despite controlling for health behaviours (Model 3: RR = 1.29; 95%CI = 1.04-1.60), and area-level education (Model 4: RR = 1.30; 95%CI = 1.05-1.62). In the lowest income group, having a bachelor’s degree was significantly associated with a 2.73 times higher risk of developing MetS (Model 4: RR = 2.73; 95%CI = 1.23-6.06).

## Discussion

As far as we are aware, this is the first longitudinal study to document a relationship between area-level SEP (measured as education and income) and incident MetS. In this well-defined Australian cohort, incident MetS occurred in 18.7% of men and 13.1% of women after an average of 3.6 years follow up. These data are comparable to similar statistics in other western countries (e.g., United States (US) [61,62]), but higher than in some Asian populations (e.g., Korean [63], Japan [64], or Taiwan [65]), though incidence estimates are subject to different definitions of MetS [66]. Area-level education was independently and inversely associated with the incident MetS. Men and women living in areas where a greater proportion of the population complete a university education, independent of their own income, education, and health risk behaviours, were significantly less likely to develop MetS than their counterparts in areas where a lower proportion of the population obtain this level of education. The association between area-level income and the incident MetS, on the other hand, was modified by individual-level income in which a statistically significant association was only observed for the high income participants. These component-specific findings highlight the importance of investigating separate effects of specific features of the area-level SEP on the occurrence of MetS.

Our observation of a relationship between area-level education and incident MetS is plausible and consistent with the results of earlier studies investigating cardiometabolic risk factors. Higher neighbourhood education, for example, has in past studies been reported to be significantly associated with lower body mass index (BMI), and a lower prevalence of overweight/obesity [67], and hypertension [68]. The absence of a statistically significant association with individual-level income and education in multivariate regression models suggests that the ability of area-level education to predict incident MetS was robust, over and above the predictive ability of individual-level SEP. With each percentage change in the proportion of individuals with a university education resulting in a 2% difference in the risk of acquiring MetS, socioeconomic disparities in the development of MetS could be substantial, given the marked differentials in the distribution of population with university education across Suburbs in the study region (i.e., from 0% to 21% of the population).

Several mechanisms can be proposed to explain the protective effect of area-level education on the incidence of MetS. First, a greater proportion of individuals with a higher level of education in communities can be plausibly linked to uptake of rapid dissemination of health education messages regarding cardiometabolic health such as healthy dietary behaviours and physical activity, both protective against metabolic disorders and the MetS. It is likely that such protective behaviours are quickly diffused throughout communities where a large proportion of residents have a higher level of education, leading to the establishment of social norms affecting health behaviours of even the less educated in these communities. Second, it is possible that communities with greater proportions of highly educated individuals are more aware of the impact of the residential environment on their health and thus are able to invest additional resources to establish and/or maintain a healthful living environment. Complementary literature indicates that neighbourhood education is positively associated with greater neighbourhood walkability [68] and availability of healthy foods [68,69], which in turn, can encourage physical activity and healthy diet. Third, it has been postulated that highly educated individuals, who often have a high level of health literacy, tend to cluster in areas with advantaged social environments. For instance, earlier studies have reported a positive association between neighbourhood education and greater neighbourhood safety and social cohesion (e.g., [68]). As a result, the sources of chronic stress (e.g., poor social cohesion, violence, or crime) that induce metabolic abnormalities and MetS through endocrine pathways [46,70,71] would be less likely to occur in communities where a greater proportion of local residents achieve a high level of education.

In examining the relationships between area-level income and the incident MetS, no statistically significant association was found in models involving the entire sample. For the highest income participants, however, area-level income was positively, rather than negatively, associated with the occurrence of MetS. Such findings were not supportive of the proposed hypotheses and mirror the current debate on the relationship between income and health, with some arguing that area-level income is not associated with individual health outcomes [72-74]. However the counterintuitive finding as seen in the high-income participants is not without precedent. In a US-based study, for example, a positive association between neighbourhood socioeconomic advantage and a worsening insulin resistance syndrome profile was reported for black men, but not for other ethnic groups [46]. In another study, area-level income was not significantly associated with BMI in black men and black women, while a higher individual-level income was positively associated with increased BMI in white men and black men [75]. Furthermore, it is worth commenting that a greater risk of MetS in low income people with an university education as found in our analysis could also reflect the effect of *status discrepancy*[76]. In highly educated individuals who earned a low income, there may be inner conflict over a sense of self-worth that drives undue actions and negative biological responses, leading to the increased risk for the MetS. However, this finding should be interpreted with caution given the small sample involved in the analysis and possible misreport of individual-level incomes that often occur in surveys [77].

The study has several important strengths. First, it is the first study using a longitudinal design to examine the role of area-level SEP in shaping the development of MetS in an Australian population. The observed associations, therefore, were not affected by the influence of prevalence-incidence bias and the potential for reverse causation as experienced by previous studies that relied on cross-sectional data. Second, the study overcomes shortcomings associated with the use of composite measures to rate the area-level SEP, unravelling relationships to assess the independent effect of area-level education and income on the development of MetS. Moreover, the analysis also included evaluation of individual- and area-level SEP interactive effects on the occurrence of MetS. Finally, important confounding factors (e.g., health risk behaviours) were taken into account in the analyses.

Our study is not without limitations. First, 23% loss to follow up might have caused selection bias. However, in a post-hoc analysis, the Wave 2 sample was similar to the baseline sample with respects to frequency distributions of participants’ baseline characteristics: age, sex, household income, education, and behaviours (i.e., smoking, alcohol consumption, and physical activity). Furthermore, the low participation rate among the eligible population may have also engendered selection bias. However, in a published analysis conducted after baseline recruitment to examine the cohort participants in comparison with the eligible population, it was reported that there were no major differences in terms of cardiometabolic behavioural and biological risk factors (i.e., current smoking status, physical activity, BMI, hypertension, blood cholesterol level), though study participants were more likely to be in the middle level of household income and education attainment (i.e., finishing high school) [78]. Second, socioeconomic characteristics under the study were limited to income and education, which may not capture fully the multi-faceted nature of SEP. Third, area- and individual-level socioeconomic characteristics were only measured at baseline, which was not representative of lifetime socioeconomic conditions. Furthermore, possible change to neighbourhood and individual SEP during the follow up period that also influences cardiometabolic health [79] was not accounted for in analysis. Similarly, area-level SEP measures were not updated for those who moved to a new residential area prior to the second clinical examination. As a result, assessment of area-SEP for these participants may not be entirely accurate, potentially causing some bias in the observed relationships. However, as we found that only 16% of the entire sample had changed their residential location, the effect of bias, if present, is likely to be minimal.

Fourth, as individuals can enter and exit the definition of MetS in a given time period (e.g., one year) depending on changes in levels of its clinical components [80], there is a possibility that in assessing the incident MetS, the current study has missed participants who developed MetS and then reversed prior to their second clinic visit, particularly those with a long interval between the two visits (e.g., >five years). This shortcoming may have resulted in an underestimate (not an overestimate) of the true MetS incidence and strength of its relationships with area-level SEP characteristics. Fourth, in examining associations between area- level income and the MetS incidence, stratified analysis was subject to small sample sizes and was therefore potentially under-powered to detect a significant association in low and middle income groups, while the presence of a significant association in the high income group may possibly be due to chance. Finally, the findings are potentially susceptible to the modifiable area unit problem, whereby the analytical results are sensitive to the definition of the spatial unit employed [81]. For example in this study, the operationalisation of area-level SEP at the State Suburb and reported associations with MetS incidence may be reflective of the underlying spatial properties (i.e., level of aggregation and configuration of zoning).

## Conclusions

The results of this study support that area-level SEP can operate both independently and interactively with individual-level socioeconomic factors to influence the risk of developing MetS. Higher area-level education appeared to be plausibly and persistently associated with a lower risk for developing MetS, and therefore can present a marker of a healthful living environment protecting individuals, even the less individually educated, from acquiring MetS. In future public health interventions, special efforts are required to improve area-level environments and resources to facilitate healthy lifestyle and behaviours in communities where a large proportion of local residents are less educated in order to address socioeconomic disparities in the distribution and development of MetS. Findings on the relationship between area-level income and the MetS incidence, on the other hand, were counterintuitive and may be context-specific and therefore should be further examined in future studies.

To better delineate relationships between area-level SEP and the incidence of MetS, future studies should expand investigation to evaluating the effect of other area-level SEP characteristics (e.g., occupational composition, employment status). A larger sample size will be needed to provide statistical power sufficient to assess effects across different individual SEP strata when evidence for interaction is present. In addition, regular clinical assessments during follow up are required to avoid the possibility for underreporting of incident MetS and better evaluate its relationships with area-level SEP. To understand mechanisms through which area-level SEP influences cardiometabolic risk factors and MetS, further studies are also needed to assess the role of other environmental factors such as built, social, or service environments in the risk of acquiring MetS. Together with the current study, findings from future studies will be valuable in guiding evidence-based health policy and public health interventions to reduce cardiometabolic risk factors and prevent the occurrence of MetS, and as a result reducing the risk for subsequent cardiometabolic diseases.

## Abbreviations

ABS: Australian Bureau of Statistics; AUD: Australian dollar; BMI: Body mass index; CATI: Computer-assisted interview; CI: Confidence interval; CHD: Coronary heart disease; GEE: Generalised estimating equations; HDL: High-density lipoprotein; MetS: Metabolic syndrome; NWAHS: North West Adelaide Health Study; PAMS: Place and metabolic syndrome; RR: Incidence risk ratio; SA: South Australia; SEP: Socioeconomic position; SSC: State suburb; sd: Standard deviation.

## Competing interests

The authors declare that they have no competing interests.

## Authors’ contributions

AN, CP and MD contributed to the conception and design of the study. AT and RA were responsible for the collection of the North West Adelaide Health Study data, and provided the data for analysis. CP and NH managed and created individual-level measures. NC and NH undertook extractions of geospatial information. AN undertook the data analysis and drafted the manuscript. AN, CP, NH, NC, and MD contributed to interpretation of the results. All authors provided critical insight, and revisions to the manuscript. All authors read and approved the final version of the manuscript submitted for publication.

## Pre-publication history

The pre-publication history for this paper can be accessed here:

http://www.biomedcentral.com/1471-2458/13/681/prepub

## Supplementary Material

Additional file 1 NWAH Study – Stage 1 CATI/QPL Telephone Recruitment Questionnaire. Click here for file

Additional file 2 **QUESTIONNAIRE A.** Stage 1 – Phase 1A & Phase 1B. Click here for file

Additional file 3 **CATI RECRUITMENT QUESTIONNAIRE.** NWAH STUDY STAGE 2. From 7 June 2004. Click here for file

Additional file 4 **Questionnaire B.** NWAH Study 2 - 04/05. Click here for file
