# Supplementary material for: Area-level socioeconomic characteristics and incidence of metabolic syndrome: a prospective cohort study
Source: BMC Public Health. 2013 Jul 25;13:681. doi: 10.1186/1471-2458-13-681 (PMC3733986; doi:10.1186/1471-2458-13-681)
Supplement: Additional file 1 — NWAH Study – Stage 1 CATI/QPL Telephone Recruitment Questionnaire. [file 1471-2458-13-681-S1.pdf]

NWAH Study – Stage 1
CATI / QPL Telephone Recruitment Questionnaire

A. INTRODUCTION

Good ...... My name is ...... I am phoning on behalf of the North West Adelaide Health Study at the Queen Elizabeth Hospital. We are conducting this study on a number of health issues with people living in the northern and western suburbs of Adelaide.

Could I please speak with the person in the household, aged 18 and over, who was the last to have a birthday.

Either

a) Repeat introduction – go to A.1

b) If required person not available ask for a suitable time to call back. Record first name and make appointment (End)

c) If required person incapable/ deaf/ ill/disabled

1) note reason

2) Go to E.2

d) Continue if correct person on line

A.1 We recently sent your household a letter from the Professor of Medicine about the health study. Did you receive the letter identifying the benefits of the study?

(Single Response)

1. Yes [ ] Go to B.
2. No [ ]
3. Don't know [ ]

The letter explained that one person from your household would be asked to be involved in a major health study being conducted by the Department of Medicine at the Queen Elizabeth Hospital. The letter also explained the benefits of being involved in the study.

[IF REFUSAL AT ANY STAGE GO TO SECTION E]

A.2 Would you like me to post the letter to you?

(Single Response)

1. Yes [ ] Go to A.3
2. No [ ] Go to B.

A.3 Could you please give me your name and address so that I can post you the letter?

1. Name

____

2. Address

____

____

Postcode 5 _ _ _

A.4 Correct postcode?

Postcodes for North West region

5007 – 5025, 5031 – 5033, 5037 & 5040, 5088 - 5093, 5095 - 5098, 5106 - 5117, 5120 - 5121, 5125 - 5127

5035, 5037, 5038, 5082, 5094, 5118 and in these suburbs
Ashford, Camden Park, Cavan, Dry Creek, Gawler, Gawler East, Gawler South, Gawler West, Glandore, Keswick, Keswick Terminal, Kurralta Park, Netley, North Plympton, Ovingham, Plympton, Plympton North, Willaston

(Single Response)

1. Yes [ ] Go to D.
2. No [ ] Go to C.

B. POSTCODE

As the study will only relate to certain groups of people, could you please tell me

B.1 What is your Postcode?

(Single Response. If postcode is not known enter 5999)

5_ _ _

Sequence Guide: If B.1 < 5999 Go to B.3

B.2 What suburb do you live in?

(Single Response. Enter suburb)

____

B.3 Correct postcode?

Postcodes for North West region

5007 – 5025, 5031 – 5033, 5037 & 5040, 5088 - 5093, 5095 - 5098, 5106 - 5117, 5120 - 5121, 5125 - 5127

5035, 5037, 5038, 5082, 5094, 5118 and in these suburbs
Ashford, Camden Park, Cavan, Dry Creek, Gawler, Gawler East, Gawler South, Gawler West, Glandore, Keswick, Keswick Terminal, Kurralta Park, Netley, North Plympton, Ovingham, Plympton, Plympton North, Willaston

(Single Response)

1. Yes [ ] Go to D.
2. No [ ]

C. TERMINATE

It is important that we speak to somebody who lives in the North West area of Adelaide. Unfortunately, that excludes you from the study – thank you very much for your time.

D. RECRUITMENT

D.1 As was mentioned in the letter, we are asking selected people to be involved in this very important study. This includes a health assessment. This will tell us more about people in the region and will also benefit the individuals who participate. The study involves attending The Queen Elizabeth Hospital or the Lyell McEwin Health Service clinic for a health assessment and a fasting blood test.

D.2 This study will benefit you and your community. All efforts will be made to make your involvement as convenient as possible for you. Would you have any objection to taking part in this very important study?

(Single Response)

1. No objection [ ] Go to D.6
2. Yes, have objection [ ]
3. Depends [ ]
4. Don’t know [ ]

D.3 Could I ask your reason for not wanting to be involved?

(Single Response)

1. Too old [ ]
2. Too sick [ ]
3. No health problem [ ]
4. Too busy [ ]
5. Don’t want to participate [ ]
6. Other (specify) [ ]
7. No [ ] Go to E.1

D.4 Try to convert. Successful?

(Single Response)

1. Yes [ ] Go to D.6
2. No (but a maybe) [ ] Go to E.2
3. No – definite [ ] Go to E.2

D.5 Included in Phase 1A only
You are still very important to us because you are a representative of the North West area. Can I ask the study coordinator to speak with you and try to find a way to help you be involved?
(Single Response)

1. Yes - contact [ ] Go to D.11
2. Yes - consent [ ] Go to D.6
3. No [ ] Go to E.2

D.6 **I now need to make an appointment for you to attend The Queen Elizabeth Hospital or the Lyell McEwin Health Service clinic. This appointment will take about 40 minutes at a time between 7.30 and 11.30am. We will require you to have a fasting blood test.** Deleted from Phase 1A: offer to have appt at either IMVS or clinic and responses related to these choices (TQEH & LmcE)

D.7 Sequence guide: address previously taken? (If A.2=1).

(Single Response)
1. Yes [ ] Go to D.8
2. No [ ] Go to D.10

D.8 We will send out detailed information about the study to the address you previously gave me.

D.9 Sequence guide: Go to D.11

D.10 Could I have your name and address so we (deleted: the study co-ordinator) **can send you more detailed information about the study?**

1. Name
____
2. Address
____
____

Postcode 5 _ _ _

D.11 Is this the best phone number to contact you, or is there another number (eg. work, mobile) that might be more appropriate?

(Multiple Response)
1. This is best number [ ]
2. Other number _____
Details ________________
3. Other number _____
Details ________________

D.12 Can I just confirm this phone number?

1. Number _____

D.13 Sequence guide: go to Section F.

E. REFUSAL DEMOGRAPHICS

E.1 Could we just ask why you do not want this household to be included in the study?

(Single Response)
1. Too busy [ ]
2. Worried, who you are [ ]
3. Don't like surveys [ ]
4. Invasion of privacy [ ]
5. Other (specify) [ ]
6. Refused [ ]

E.2 We need to know some information about the people who are not included in the study. What age group are/is you/that person (the person with the next birthday) in?

(Read options, Single Response, code an approximate category if refused)
1. **18-24 years** [ ]
2. **25-29 years** [ ]
3. **30-34 years** [ ]
4. **35-39 years** [ ]
5. **40-44 years** [ ]
6. **45-49 years** [ ]
7. **50-54 years** [ ]
8. **55-59 years** [ ]
9. **60-64 years** [ ]
10. **65-69 years** [ ]
11. **70-74 years** [ ]
12. **75 years or over** [ ]
13. Other - Specify [ ]

E.3 Gender (or Gender)

(Single Response)
1. Male [ ]
2. Female [ ]

E.4 Is this the person randomly selected (ie last birthday)?

(Single Response)
1. Yes [ ]
2. Definitely not [ ] Go to H.4
3. Unknown [ ] Go to H.4

F. HEALTH CONDITIONS

F.1 I would like to ask you a few questions about your health. Have you ever been told by a doctor that you have any of the following conditions?

(Read Options. Multiple Response)

1. **Diabetes** [ ] *medication
2. **Asthma** [ ] *medication
3. **Bronchitis** [ ]
4. **Emphysema** [ ]
5. **Heart attack** [ ]
6. **Stroke** [ ]
7. **Angina** [ ]
8. None [ ]

G. RISK FACTORS

G.1 Do you currently smoke?

(Single Response)

1. Yes [ ] Go to G.3
2. No [ ]
3. Occasionally [ ]

G.2 Have you ever smoked regularly (that is, at least once a day)?

(Single Response)

1. Yes [ ]
2. No [ ]

G.3 Phase 1B only
Have you ever been told by a doctor or nurse that you have high cholesterol?

(Single Response)

1. Yes [ ]
2. No [ ] Go to G.5
3. Don't know [ ] Go to G.5
4. Never measured [ ] Go to G.5

G.4 Phase 1B only
Do you still have high cholesterol?

(Single Response)

1. Yes [ ]
2. No [ ]
3. Don't know [ ]

G.5 Phase 1B only
Have you ever been told by a doctor or nurse that you have high blood pressure?

(Single Response)

1. Yes [ ]
2. No [ ] Go to G.7
3. Don't know [ ] Go to G.7
4. Never measured [ ] Go to G.7

G.6 Phase 1B only
Do you still have high blood pressure?

(Single Response)

1. Yes [ ]
2. No [ ]
3. Don't know [ ]

G.7 Phase 1B only
What is your height without shoes?

(Single Response)

1. Centimetres ______
2. Feet: inches ______
3. Don't know [ ]

G.8 Phase 1B only
What is your weight? (undressed in the morning)

(Single Response)

1. Kilograms [ ]
2. Stones: pounds [ ]
3. Don't know [ ]

F. HEALTH CONDITIONS

F2 In the last 12 months have you been told by a doctor that you have any of the following conditions?

(Read Options. Multiple Response)

1. **Anxiety** [ ]
2. **Depression** [ ]
3. **A stress related problem** [ ]
4. **Any other mental health problem** [ ]
5. None [ ]

Sequence guide : If not recruited (refusal demographics collected E.3 = 1 or 2 Go to H.3

H. DEMOGRAPHICS

Now to finish with some general questions.

H.1 How old are you?

(Single Response. Enter 999 if not stated)

______

H.2 Voice (ask if unsure)

(Single Response)

1. Male [ ]
2. Female [ ]

H.3 What kind of work have you done for most of your life?

(Single Response)

1. Specify _____

H.4 Including yourself how many people aged 18 and over live in this household?

(Single Response. Enter number of people 18 years and over)

[ ]

H.5 How many children under 18 years live in your household?

(Single Response. Enter number of people under 18 years. Enter 0 if none)

[ ]

H.6 That concludes the interview. On behalf of the North West Adelaide Health Study, thank you very much for your time.

(Interviewer note any other comments about the interview)

____

____

____

H.7 Date of interview

H.8 Day of week interview undertaken
