# Supplementary material for: Area-level socioeconomic characteristics and incidence of metabolic syndrome: a prospective cohort study
Source: BMC Public Health. 2013 Jul 25;13:681. doi: 10.1186/1471-2458-13-681 (PMC3733986; doi:10.1186/1471-2458-13-681)
Supplement: Additional file 2 — QUESTIONNAIRE A. Stage 1 – Phase 1A & Phase 1B. [file 1471-2458-13-681-S2.pdf]

QUESTIONNAIRE A

Stage 1 – Phase 1A & Phase 1B

Please read the following instructions before answering the questions.

1. Please complete all the questions as per instructions by placing a tick in the box that most closely corresponds to your answer.
2. Your answers will remain strictly confidential. Results of the study may be published in a medical journal but no information that may lead to the identification of any individual will be released.
3. This questionnaire should take approximately 15 minutes to complete.
4. If you have any problems, please contact the clinic coordinator on (tel) 8222 7866.
5. When you have completed the questionnaire, please bring it with you to your appointment at The Queen Elizabeth Hospital or the Lyell McEwin Health Service.

A. GENERAL HEALTH AND WELL BEING

These first questions ask for your views about your health, how you feel and how well you are able to do your usual activities. Please answer each question.

A.1 In general would you say your health is: *(tick one box only)*

- ❑ 1 Excellent
- ❑ 2 Very good
- ❑ 3 Good
- ❑ 4 Fair
- ❑ 5 Poor

A.2 Compared to one year ago, how would you rate your health in general now? *(tick one box only)*

- ❑ 1 Much better now than one year ago
- ❑ 2 Somewhat better now than one year ago
- ❑ 3 About the same as one year ago
- ❑ 4 Somewhat worse now than one year ago
- ❑ 5 Much worse now than one year ago

The next questions relate to activities you might do during a typical day. Please tell us if your health now limits you a lot, limits you a little or does not limit you at all in these activities.

A.3 Vigorous activities, such as running, lifting heavy objects, participating in strenuous sports? *(tick one box only)*

- ❑ 1 Yes, limited a lot
- ❑ 2 Yes, limited a little
- ❑ 3 No, not limited at all

A.4 Moderate activities, such as moving a table, pushing a vacuum cleaner, bowling or playing golf? *(tick one box only)*

- ❑ 1 Yes, limited a lot
- ❑ 2 Yes, limited a little
- ❑ 3 No, not limited at all

A.5 Lifting or carrying groceries? *(tick one box only)*

- ❑ 1 Yes, limited a lot
- ❑ 2 Yes, limited a little
- ❑ 3 No, not limited at all

A.6 Climbing several flights of stairs? *(tick one box only)*

- ❑ 1 Yes, limited a lot
- ❑ 2 Yes, limited a little
- ❑ 3 No, not limited at all *(if No…go to A.8)*

A.7 Climbing one flight of stairs? *(tick one box only)*

- ❑ 1 Yes, limited a lot
- ❑ 2 Yes, limited a little
- ❑ 3 No, not limited at all

A.8 Bending, kneeling or stooping? *(tick one box only)*

- ❑ 1 Yes, limited a lot
- ❑ 2 Yes, limited a little
- ❑ 3 No, not limited at all

A.9 Walking more than one kilometre? *(tick one box only)*

- ❑ 1 Yes, limited a lot
- ❑ 2 Yes, limited a little
- ❑ 3 No, not limited at all *(if No…go to A.12)*

A.10 Walking half a kilometre? *(tick one box only)*

- ❑ 1 Yes, limited a lot
- ❑ 2 Yes, limited a little
- ❑ 3 No, not limited at all *(if No…go to A.12)*

A.11 Walking 100 metres? *(tick one box only)*

- ❑ 1 Yes, limited a lot
- ❑ 2 Yes, limited a little
- ❑ 3 No, not limited at all

A.12 Bathing or dressing yourself? *(tick one box only)*

- ❑ 1 Yes, limited a lot
- ❑ 2 Yes, limited a little
- ❑ 3 No, not limited at all

The following four questions ask you about your physical health and your daily activities.

During the <u>last four weeks</u> have you ....

A.13 Had to cut down on the amount of time you spent on work or other activities as a result of your physical health? *(tick one box only)*

❑ 1 Yes
❑ 2 No

A.14 Accomplished less than you would like as a result of your physical health? *(tick one box only)*

❑ 1 Yes
❑ 2 No

A.15 Been limited in the kind of work or other activities as a result of your physical health? *(tick one box only)*

❑ 1 Yes
❑ 2 No

A.16 Had difficulty performing the work or other activities as a result of your physical health (for example, it took extra effort)? *(tick one box only)*

❑ 1 Yes
❑ 2 No

The following three questions ask you about your emotions and your daily activities.

During the <u>past four weeks</u> have you ....

A.17 Had to cut down on the amount of time you spent on work or other activities as a result of any emotional problems such as feeling depressed or anxious? *(tick one box only)*

❑ 1 Yes
❑ 2 No

A.18 Accomplished less than you would like as a result of any emotional problems? *(tick one box only)*

❑ 1 Yes
❑ 2 No

A.19 Had to not do work or other activities as carefully as usual as a result of any emotional problems? *(tick one box only)*

❑ 1 Yes
❑ 2 No

A.20 During the past four weeks, to what extent has your physical health or emotional problems interfered with your normal social activities with family, friends, neighbours or groups? Has it interfered: *(tick one box only)*

❑ 1 Not at all
❑ 2 Slightly
❑ 3 Moderately
❑ 4 Quite a bit
❑ 5 Extremely

A.21 How much bodily pain have you had during the past four weeks? *(tick one box only)*

❑ 1 None *(if None…go to A.23) PTO* →
❑ 2 Very mild
❑ 3 Mild
❑ 4 Moderate
❑ 5 Severe
❑ 6 Very severe

A.22 During the past four weeks, how much did pain interfere with your normal work (including both work outside the home and housework)? *(tick one box only)*

❑ 1 Not at all
❑ 2 A little bit
❑ 3 Moderately
❑ 4 Quite a bit
❑ 5 Extremely

These questions are about how you feel and how things have been with you during the past four weeks. For each question please give the one answer that comes closest to the way you have been feeling.

How much during the past four weeks:

A.23 Did you feel full of life? *(tick one box only)*

- ❑ 1 All the time
- ❑ 2 Most of the time
- ❑ 3 A good bit of the time
- ❑ 4 Some of the time
- ❑ 5 A little of the time
- ❑ 6 None of the time

A.24 Have you been a very nervous person? *(tick one box only)*

- ❑ 1 All the time
- ❑ 2 Most of the time
- ❑ 3 A good bit of the time
- ❑ 4 Some of the time
- ❑ 5 A little of the time
- ❑ 6 None of the time

A.25 Have you felt so down in the dumps that nothing could cheer you up? *(tick one box only)*

- ❑ 1 All the time
- ❑ 2 Most of the time
- ❑ 3 A good bit of the time
- ❑ 4 Some of the time
- ❑ 5 A little of the time
- ❑ 6 None of the time

A.26 Have you felt calm and peaceful? *(tick one box only)*

- ❑ 1 All the time
- ❑ 2 Most of the time
- ❑ 3 A good bit of the time
- ❑ 4 Some of the time
- ❑ 5 A little of the time
- ❑ 6 None of the time

Did you have a lot of energy? *(tick one box only)*

- ❑ 1 All the time
- ❑ 2 Most of the time
- ❑ 3 A good bit of the time
- ❑ 4 Some of the time
- ❑ 5 A little of the time
- ❑ 6 None of the time

A.27 Have you felt down? *(tick one box only)*

- ❑ 1 All the time
- ❑ 2 Most of the time
- ❑ 3 A good bit of the time
- ❑ 4 Some of the time
- ❑ 5 A little of the time
- ❑ 6 None of the time

A.28 Did you feel worn out? *(tick one box only)*

- ❑ 1 All the time
- ❑ 2 Most of the time
- ❑ 3 A good bit of the time
- ❑ 4 Some of the time
- ❑ 5 A little of the time
- ❑ 6 None of the time

A.29 Have you been a happy person? *(tick one box only)*

- ❑ 1 All the time
- ❑ 2 Most of the time
- ❑ 3 A good bit of the time
- ❑ 4 Some of the time
- ❑ 5 A little of the time
- ❑ 6 None of the time

A.30 Did you feel tired? *(tick one box only)*

- ❑ 1 All the time
- ❑ 2 Most of the time
- ❑ 3 A good bit of the time
- ❑ 4 Some of the time
- ❑ 5 A little of the time
- ❑ 6 None of the time

A.31 During the past four weeks, how much of the time has your physical health or emotional problems interfered with your social activities (like visiting friends, relatives, etc)? *(tick one box only)*

- ❑ 1 All the time
- ❑ 2 Most of the time
- ❑ 3 A good bit of the time
- ❑ 4 Some of the time
- ❑ 5 A little of the time
- ❑ 6 None of the time

How true or false is each of the following statements for you?

A.32 "I seem to get sick a little easier than other people." *(tick one box only)*

- ❑ 1 Definitely true
- ❑ 2 Mostly true
- ❑ 3 Don't know
- ❑ 4 Mostly false
- ❑ 5 Definitely false

A.33 "I am as healthy as anybody I know." *(tick one box only)*

- ❑ 1 Definitely true
- ❑ 2 Mostly true
- ❑ 3 Don't know
- ❑ 4 Mostly false
- ❑ 5 Definitely false

A.34 "I expect my health to get worse." *(tick one box only)*

- ❑ 1 Definitely true
- ❑ 2 Mostly true
- ❑ 3 Don't know
- ❑ 4 Mostly false
- ❑ 5 Definitely false

A.35 "My health is excellent." *(tick one box only)*

- ❑ 1 Definitely true
- ❑ 2 Mostly true
- ❑ 3 Don't know
- ❑ 4 Mostly false
- ❑ 5 Definitely false

B. EXERCISE

The next questions are about exercise you may do for sport, recreation or fitness.

B.1 In the last two weeks, did you do any walking for sport, recreation or fitness? *(tick one box only)*

- ❑ 1 Yes
- ❑ 2 No *(If no…go to B.4)*

B.2 How many times did you do any walking for exercise in the last two weeks?

1 Enter number of times __________

❑ 99 Don't know

B.3 What was the total amount of time you spent walking in the last two weeks?

1 Enter number of hours __________

2 Enter number of minutes __________

B.4 In the last 2 weeks, (apart from walking) did you do any exercise which caused a moderate increase in your heart rate or breathing? *(tick one box only)*

- ❑ 1 Yes
- ❑ 2 No *(If no…go to B.7)*

B.5 How many times did you do any moderate exercise in the last two weeks?

1 Enter number of times __________

❑ 99 Don't know

B.6 What was the total amount of time you spent doing moderate exercise in the last two weeks?

1 Enter number of hours __________

2 Enter number of minutes __________

B.7 In the last 2 weeks, did you do any other exercise which caused a large increase in your heart rate or breathing, that is, vigorous exercise? *(tick one box only)*

- ❑ 1 Yes
- ❑ 2 No *(If no…go to* C.1)

PTO

B.8 How many times did you do any vigorous exercise in the last two weeks?

1 Enter number of times __________

☐ 99 Don't know

B.9 What was the total amount of time you spent doing vigorous exercise in the last two weeks?

1 Enter number of hours __________

2 Enter number of minutes __________

C. HEALTH CARE UTILISATION

C.1 How many times in the last 12 months have you used these health services in South Australia?
(Enter number of times for each service used. If unsure, approximate number will do.)

1 General practitioner __________

2 Community health centre __________

3 District nurses or other community nurses __________

4 Psychologist __________

5 Psychiatrist __________

6 Day surgery __________

7 Hospital – Accident & Emergency Department __________

8 Hospital – Clinic (outpatient/ specialist/allied health) __________

9 Eye specialist/ ophthalmologist __________

10 Other specialist doctor (not in a hospital) __________

11 Physiotherapist __________

12 Chiropractor __________

13 Alternative therapist (eg. naturopath, osteopath) __________

14 Podiatrist __________

15 Dietician __________

16 Nurse educator __________

17 Other *(please specify)*

____________ __________

____________ __________

____________ __________

D. FAMILY HISTORY

D.1 Do, or did, any of your relatives have diabetes? (Blood/first degree relations only) *(tick all that apply)*

☐ 1 Mother
☐ 2 Father
☐ 3 Sister
☐ 4 Brother
☐ 5 Grandmother
☐ 6 Grandfather
☐ 7 Other *(please specify*__________)
☐ 8 No
☐ 9 Don't know

D.2 Do, or did, any of your relatives have heart disease, for example, heart attack or heart failure? (Blood/first degree relations only) *(tick all that apply)*

☐ 1 Mother
☐ 2 Father
☐ 3 Sister
☐ 4 Brother
☐ 5 Grandmother
☐ 6 Grandfather
☐ 7 Other *(please specify)* __________
☐ 8 No
☐ 9 Don't know

D.3 Have any of your relatives ever had a stroke? (Blood/first degree relations only) *(tick all that apply)*

☐ 1 Mother
☐ 2 Father
☐ 3 Sister
☐ 4 Brother
☐ 5 Grandmother
☐ 6 Grandfather
☐ 7 Other *(please specify)* __________
☐ 8 No
☐ 9 Don't know

E. DIABETES AND HIGH BLOOD SUGAR

E.1 Have you ever been told by a doctor that you have diabetes? *(tick one box only)*
(Phase 1A only: Yes / No (If no, go to E3) – females to please answer E2, males to please answer E3).

- ❑ 1 Yes *(If male…go to E.4)*
  (If female …go to E.2)
- ❑ 2 No *(If no…go to* E.3*)*

For women who said "yes" to question e1:

E.2 Were you pregnant when you were first told you had diabetes? *(tick one box only)*

- ❑ 1 Yes
- ❑ 2 No

E.3 Have you ever been told by a doctor that you have high blood sugar or a touch of sugar? *(tick one box only)*

- ❑ 1 Yes
- ❑ 2 No

E.4 Have you got diabetes or high blood sugar now? *(tick one box only)*

- ❑ 1 Yes
- ❑ 2 No *(If no…go to* F.1*)* → *next column*

E.5 Other than gestational diabetes, what type of diabetes were you told you had? *(tick one box only)*

- ❑ 1 Type 1 – Insulin dependent – Juvenile onset
- ❑ 2 Type 2 – Non-insulin dependent – Mature onset
- ❑ 3 Don't know
- ❑ 4 Other *(please specify)* ___________

E.6 (Phase 1B only) **Other than gestational diabetes, when were you first told you had diabetes or high blood sugar?** *(tick one box only)*

- ❑ 1 Within the last twelve months
- ❑ 2 1 to 2 years ago
- ❑ 3 3 to 5 years ago
- ❑ 4 6 to 10 years ago
- ❑ 5 More than 10 years ago
- ❑ 6 Don't know

F. ASTHMA

F.1 Have you ever had asthma?
(tick one box only)

- ❑ 1 Yes
- ❑ 2 No *(If no…go to F.4)*

F.2 Was your asthma confirmed by a doctor?
(tick one box only)

- ❑ 1 Yes
- ❑ 2 No *(If no…go to* F.4*)*
- ❑ 3 Don't know *(If don't know…go to* F.4*)*

F.3 Do you still have asthma?
(tick one box only)

- ❑ 1 Yes
- ❑ 2 No
- ❑ 3 Don't know

F.3A (Phase 1B only) **When were you first told you had asthma?**
(tick one box only)

- ❑ 1 Within the last twelve months
- ❑ 2 1 to 2 years ago
- ❑ 3 3 to 5 years ago
- ❑ 4 6 to 10 years ago
- ❑ 5 More than 10 years ago
- ❑ 6 Don't know

F.3B (Phase 1B only) **How do you rate your asthma severity?**
(tick one box only)

- ❑ 1 Not a problem
- ❑ 2 Mild
- ❑ 3 Moderate
- ❑ 4 Severe

BRONCHITIS

F.4 Have you ever had bronchitis?
(tick one box only)

- ❑ 1 Yes
- ❑ 2 No *(If no…go to* F.7*)* → *PTO*
- ❑ 3 Don't know *(If don't know…go to* F.7*)* → *PTO*

F.5 Was your bronchitis ever confirmed by a doctor? *(tick one box only)*

- ❑ 1 Yes
- ❑ 2 No *(If no…go to* F.7*)*
- ❑ 3 Don't know *(If don't know…go to* F.7*)*

F.6 (Phase 1B only) **How often do you have bronchitis?** *(tick one box only)*

- ❑ 1 Less than once a year
- ❑ 2 Once a year
- ❑ 3 More often than once a year

F.6A (Phase 1B only) **When were you first told you had bronchitis?** *(tick one box only)*

- ❑ 1 Within the last twelve months
- ❑ 2 1 to 2 years ago
- ❑ 3 3 to 5 years ago
- ❑ 4 6 to 10 years ago
- ❑ 5 More than 10 years ago
- ❑ 6 Don't know

EMPHYSEMA

F.7 Have you ever had emphysema? *(tick one box only)*

- ❑ 1 Yes
- ❑ 2 No *(If no…go to* G.1*)* → *next column*
- ❑ 3 Don't know *(If don't know…go to* G.1*)* → *next column*

F.8 Was your emphysema ever confirmed by a doctor? *(tick one box only)*

- ❑ 1 Yes
- ❑ 2 No

F.8A (Phase 1B only) **When were you first told you had emphysema?** *(tick one box only)*

- ❑ 1 Within the last twelve months
- ❑ 2 1 to 2 years ago
- ❑ 3 3 to 5 years ago
- ❑ 4 6 to 10 years ago
- ❑ 5 More than 10 years ago

G. LUNG FUNCTION

The next series of questions relate to your lung function over the past three months.

G.1 In the past three months, during a typical day, have any of these made you short of breath? *(tick one box only)*

- ❑ 1 No activity: such as at rest, while sitting or lying down
- ❑ 2 Light activity: such as walking on level ground, shopping, washing or standing
- ❑ 3 Moderate activity: such as walking up a gradual hill, climbing less than three flights of stairs or carrying a light load on level ground
- ❑ 4 Vigorous activities: such as running, walking up a steep hill, climbing three or more flights of stairs or carrying a moderate load on level ground
- ❑ 5 Other
- ❑ 6 None *(If none…go to G.3)*

G.2 In the past three months, how often were you short of breath? *(tick one box only)*

- ❑ 1 Occasionally
- ❑ 2 Most days
- ❑ 3 All of the time

G.3 In the past three months, did any of these make you wheeze? *(tick one box only)*

- ❑ 1 No activity: such as at rest, while sitting or lying down
- ❑ 2 Light activity: such as walking on level ground, shopping, washing or standing
- ❑ 3 Moderate activity: such as walking up a gradual hill, climbing less than three flights of stairs or carrying a light load on level ground
- ❑ 4 Vigorous activities: such as running, walking up a steep hill, climbing three or more flights of stairs or carrying a moderate load on level ground
- ❑ 5 Other
- ❑ 6 None *(If none…go to* G.5*)* → *PTO*

G.4 In the past three months, how often did you wheeze? *(tick one box only)*

- ❑ 1 Occasionally
- ❑ 2 Most days
- ❑ 3 Always
- ❑ 4 Other (please specify) __________

G.5 In the past three months, how often did you cough? *(tick one box only)*

- ❑ 1 Never *(If never…go to* H.1*)*
- ❑ 2 Occasionally or only during a cold or flu
- ❑ 3 Most days
- ❑ 4 Every day

G.6 In the past three months when you coughed, how much sputum or phlegm did you produce? *(tick one box only)*

- ❑ 1 None
- ❑ 2 Just a little
- ❑ 3 Several tablespoons a day
- ❑ 4 A coffee cup or more a day

H. ALCOHOL

H.1 How often do you usually drink alcohol? *(tick one box only)*

- ❑ 1 I don't drink alcohol *(go to* I.1*)* → *next column*
- ❑ 2 Less than once a week
- ❑ 3 On 1 or 2 days a week
- ❑ 4 On 3 or 4 days a week
- ❑ 5 On 5 or 6 days a week
- ❑ 6 Every day

H.2 A Standard Drink is equivalent to a schooner of full strength beer, a glass of wine or a nip of spirits. On a day when you drink alcohol, how many drinks do you usually have? *(tick one box only)*

- ❑ 1 1 or 2 drinks
- ❑ 2 3 or 4 drinks
- ❑ 3 5 or 8 drinks
- ❑ 4 9 or 12 drinks
- ❑ 5 13 or 20 drinks
- ❑ 6 More than 20 drinks

I. SMOKING

I.1 Do you currently smoke? *(tick one box only)*

- ❑ 1 Yes
- ❑ 2 No *(If no…go to* I.3*)*
- ❑ 3 Occasionally *(If occasionally…go to* I.3*)*

I.2 How many cigarettes do you usually smoke a day?

- 1 Enter number of cigarettes __________ *(go to* I.6*)*
- ❑ 2 Less than one *(go to* I.6*)*
- ❑ 3 Only smoke cigars or pipes *(go to* I.6*)*

I.3 Have you ever smoked regularly (that is, at least once a day)? *(tick one box only)*

- ❑ 1 Yes
- ❑ 2 No *(If no…go to* K. *)* → *PTO*

I.4 How many cigarettes did you usually smoke a day?

- 1 Enter number of cigarettes __________
- ❑ 2 Less than one
- ❑ 3 Only smoke cigars or pipes

I.5 How old were you when you last gave up smoking?

- 1 Enter age ___________
- ❑ 2 Can't remember

I.6 At what age did you first start smoking daily?

- 1 Enter age __________
- ❑ 2 Can't remember

K. DEMOGRAPHICS

I.7 How old were you when you left school? *(tick one box only)*

1 Enter age __________
- ❑ 2 Can't remember
- ❑ 3 Still at school *(If still at school…go to* I.10*)*

I.8 Since leaving school have you obtained a trade qualification, certificate, diploma or any other qualification? *(tick one box only)*

- ❑ 1 Yes
- ❑ 2 No *(If no…go to* I.10*)*
- ❑ 3 Don't know

I.9 What is your highest qualification? *(tick one box only)*

- ❑ 1 Bachelor degree or higher
- ❑ 2 Trade / Apprenticeship
- ❑ 3 Certificate / Diploma
- ❑ 4 Other *(please specify)* __________
- ❑ 5 Don't know

I.10 What is the approximate annual gross income of your household? (that is, for all people in the household before tax is taken out) *(tick one box only)*

- ❑ 1 Up to $12,000
- ❑ 2 $12,001 - $20,000
- ❑ 3 $20,001 - $30,000
- ❑ 4 $30,001 - $40,000
- ❑ 5 $40,001 - $50,000
- ❑ 6 $50,001 - $60,000
- ❑ 7 $60,001 - $80,000
- ❑ 8 More than $80,000

I.11 What is your country of birth? *(tick one box only)*

- ❑ 1 Australia *(If Australia…go to* I.13*)*
- ❑ 2 Austria
- ❑ 3 Bosnia-Herzegovina
- ❑ 4 Canada
- ❑ 5 China
- ❑ 6 Croatia
- ❑ 7 France
- ❑ 8 Germany
- ❑ 9 Greece
- ❑ 10 Holland / Netherlands
- ❑ 11 Hong Kong
- ❑ 12 Iran
- ❑ 13 Italy
- ❑ 14 Japan
- ❑ 15 Malaysia
- ❑ 16 New Zealand
- ❑ 17 Philippines
- ❑ 18 Poland
- ❑ 19 Slovenia
- ❑ 20 Spain
- ❑ 21 U.K. and Ireland
- ❑ 22 USA
- ❑ 23 Vietnam
- ❑ 24 Former Yugoslav Republic of Macedonia
- ❑ 25 Former Yugoslav Republics of Serbia & Montenegro
- ❑ 26 Other *(please specify)* __________

I.12 What year did you arrive in Australia?

1 Enter year ______ (*Go to* I.14) → *PTO*
- ❑ 99 Don't know (*Go to* I.14) → *PTO*

I.13 Are you of Aboriginal or Torres Strait Islander origin? (For persons of both Aboriginal and Torres Strait Islander origin, tick both 'Yes' boxes)

- ❑ 1 No
- ❑ 2 Yes, Aboriginal
- ❑ 3 Yes, Torres Strait Islander

I.14 What is your marital status?
(tick one box only)

- ❑ 1 Married or living with a partner
- ❑ 2 Separated / Divorced
- ❑ 3 Widowed
- ❑ 4 Never married

I.15 What is your work status?
(tick one box only)

- ❑ 1 Full time employed
- ❑ 2 Part time / casual employment
- ❑ 3 Unemployed
- ❑ 4 Home duties
- ❑ 5 Retired
- ❑ 6 Student
- ❑ 7 Other *(please specify)* ___________

I.16 Do you receive a pension or benefit from the Department of Social Security? (this does not include family allowance)
(tick one box only)

- ❑ 1 Yes
- ❑ 2 No
- ❑ 3 Don't know

I.17 How old are you?

1 Enter age (years) ___________

I.18 What is your postcode?

1 Enter postcode 5 __ __ __

That concludes the survey.
Thank you very much for your time.

Please make sure that you have answered all the questions.
You can return this questionnaire when you attend your clinic appointment.

If you have any problems or questions in completing this questionnaire, please telephone the Clinic Co-ordinator, on 8222 7866.
