# Supplementary material for: Area-level socioeconomic characteristics and incidence of metabolic syndrome: a prospective cohort study
Source: BMC Public Health. 2013 Jul 25;13:681. doi: 10.1186/1471-2458-13-681 (PMC3733986; doi:10.1186/1471-2458-13-681)
Supplement: Additional file 3 — CATI RECRUITMENT QUESTIONNAIRE. NWAH STUDY STAGE 2. From 7 June 2004. [file 1471-2458-13-681-S3.pdf]

CATI RECRUITMENT QUESTIONNAIRE

NWAH STUDY STAGE 2

From 7 June 2004

Good ..... My name is ..... I am phoning on behalf of the North West Adelaide Health Study.

Could I please speak with *(name of study participant*)?

Either

a) Repeat introduction – go to A.1.

b) If required person not available, ask for a suitable time to call back. Record first name and make appointment to call back (End).

c) If required person died, go to A.22. If information offered re cause of death or date, record same.

d) If required person is no longer at this telephone number, go to A.24 to record details.

A.1 We recently sent your household a letter from the Professor of Medicine about your continued participation in the North West Adelaide Health Study. Did you receive the letter identifying the benefits of continuing to help us with this study?

(Single response)

1. Yes [ ]
2. No [ ]
3. Don't know [ ]

Sequence guide: If A.1 = 1, go to A.5.

The letter explained that we would like you to return to the clinic at either The Queen Elizabeth Hospital or Lyell McEwin Health Service for the next assessment of your health.

(Interviewer note: ask participant if they would like the letter read out to them)

A.2 Would you like me to post the letter to you?

(Single response)

1. Yes [ ]
2. No [ ]

Sequence Guide If A.2 = 2, Go to A.5.

A.3 Can I please confirm that your address is ….. (read details from database)

(Multiple response)

1. Address confirmed [ ]
2. Change of address [ ]
3. Additional postal address [ ]

Sequence guide: If A.3 = 2, Go to A.4

If A.3 = 3, Go to A4a

Else go to A.5.

A.4 Change of address

(Multiple Response*. Interviewer note: Enter details.*)

1. *Enter Address* _____
2. *Enter Postcode* _ _ _ _

Sequence guide: If A.3 = 3, Go to A4a

Else go to A.5.

A4a Postal address

(Multiple Response*. Interviewer note: Enter details.*)

1. *Enter Address* _____
2. *Enter Postcode* _ _ _ _

A.5 We are calling you today as part of the second major stage of the study. This involves answering some questions on the phone now, as well as filling in a questionnaire that we will be posting out to you and also coming back to the clinic for another health assessment. All efforts will be made to make your involvement as convenient as possible for you. Would you have any objection to again taking part in this very important study?

(Single response)

1. No objection [ ]
2. Yes, have objection [ ]

Sequence guide: If A.5 = 1, go to A.12.

A.6 Your ongoing participation is vital to the success of the study in its efforts to improve the health of people both in your community and South Australia in general. Could I ask why you no longer want to be involved in the study?

(Single response)

1. Too old [ ]
2. Too sick [ ]
3. Health problem(s) [ ]
4. Too busy [ ]
5. Don't want to participate [ ]
6. Tired of being asked [ ]
7. Moved out of area [ ]
8. Moved interstate/overseas [ ]
9. Other (*specify*) [ ]
10. No [ ]
11. Currently pregnant [ ]

Sequence guide: If A.6 = 7, go to A.8.

If A.6 = 8, go to A.9.

If A.6 = 11, go to A.14

A.7 Try to convert for this stage (Stage 2). Successful?

(Single response)

1. Yes [ ]
2. No – definite [ ]

Sequence guide: If A.7 = 1, go to A.12.

if A.7 = 2, go to A.10.

A.8 (moved out of northern or western region) **You are very important to us because you are still a part of the North West Adelaide Health Study.** Try to convert - successful?

(Single response)

1. Yes [ ]
2. No – definite [ ]

Sequence guide: If A.8 = 1, go to A.12.

If A.8 = 2, go to A.10.

A.9 (interstate/overseas participants) **You are very important to us because you are still a part of the North West Adelaide Health Study. Can we send you a letter shortly, confirming the importance of your ongoing involvement in the study and asking that you contact us when you next visit Adelaide.**

(Single response)

1. Yes, will participate [ ]
2. No - definite withdrawal [ ]

Sequence guide: If A.9 = 2, go to A.23.

A.10 Would you be willing to answer a questionnaire that we would mail out?

(Single response)

1. Yes [ ]
2. No [ ]

A.11 Would you please answer some questions now, on the phone?

(Single response)

1. Yes [ ]
2. No [ ]

Sequence guide: If A.10=2 & A.11 = 2, go to A.11A

If A.10=1 & If A.3 = 1,2 (address confirmed), Go to A.19A

If A.10=2 & A.11 = 1 & If A.3 = 1,2 (address confirmed), Go to A.19

Else go to A.21

A.11A We would like to keep you up to date about the study by sending you our newsletter or invite you again to participate in any other health assessment. Would you mind if we contact you at a later date?

(Single response)

1. Yes, can contact [ ]
2. No, total withdrawal from the study [ ]

Sequence guide:

If A.11A = 1 & If A.3 = 1,2 (address confirmed), Go to A.21

If A.11A = 1 & If A.3 ≠ 1,2,3 (did not confirm address) Go to A.19

If A.11A = 2 Go to A.23.

A.12 Sequence guide: If participant is male or if participant is female aged 45 yrs and over, go to A.15.

A.13 Are you currently pregnant?

(Single response)

1. Yes [ ]
2. No [ ]

Sequence guide: If A.13 = 2, go to A.15.

A.14 Thank you - as you are currently pregnant, we won't be asking you to attend the clinic at this time. However, we would still like you to continue as part of the study cohort. Can I please ask when you are due to give birth so we can call to make a time for you to attend the clinic at a later date?

(Single response)

1. Due date: __/__/____
2. Refused 00/00/0000

Sequence guide:

If A.3 = 1,2 (address confirmed), go to A.21

Else (did not confirm address) Go to A.19.

A.15 I now need to make an appointment for you to attend The Queen Elizabeth Hospital or the Lyell McEwin Health Service clinic. This appointment will take about 1 hour at a time between 7.30 and 10.30am. We will require you to have a fasting blood test. Which clinic would you like to attend?

1. The Queen Elizabeth Hospital [ ]
2. Lyell McEwin Health Service clinic [ ]

3. Appt date: __/__/____
4. Appt time: _____

Sequence guide: If AGE $\geq$ 50 and A.15 = 1 (QEH) Go to A.16.

Else Go to A.17.

A.16 We would also like to invite you to have a test for osteoporosis, or brittle bones. This test will provide a measurement of your bone density, that is how strong your bones are. It will involve a 15 minute scan following your clinic appointment. I will be sending out more detailed information about the test in your information pack. Can I make this appointment for you?

(Single response)

1. Yes [ ]

Appt date: __/__/____ Appt time: ____

2. No [ ]
3. Don't know - will speak to clinic staff [ ]

A.17 Sequence guide: address previously taken?

If A.3 = 1,2 (address confirmed), go to A.18

Else (did not confirm address) Go to A.19A

(Single response)

1. Yes, address confirmed [ ]
2. No [ ]

A.18 We will send out an information pack to the address you have previously given me.

(Interviewer note: please explain what is being sent to them ie information about clinic procedures, a questionnaire, letter confirming appointment, a map, medication sheet)

Sequence guide: Go to A.21

A.19A [Read if necessary] **We will send out an information pack to you.**

(Interviewer note: If need to please explain what is being sent to them ie information about clinic procedures, a questionnaire, letter confirming appointment, a map, medication sheet. OR for some people just the questionnaire)

A.19 Can I please confirm that your address is ….. (read details from database)

(Multiple response)

1. Address confirmed [ ]
2. Change of address [ ]
3. Additional postal address [ ]

Sequence guide: If A.19=2 Go to A.20

If A.19=3 Go to A.20A

Else go to A.21

A.20 Change of address

1. Address ______________________
2. Postcode 5 _ _ _

Sequence guide: If A.19=3 Go to A.20A
Else go to A.21

A20A Postal address

1. Address ______________________
2. Postcode 5 _ _ _

A.21 Is this the best phone number to contact you, or is there another number (eg. work, mobile) that might be more appropriate?

(Multiple Response. Interviewer note: confirm number and determine if the best number is a home, work or other phone number)

1. This is the best number [ ]
2. Home number __________
3. Work number __________
4. Mobile number __________
5. Other number __________

A.21A Additional numbers?

(Multiple Response. Interviewer note: confirm number and determine if the best number is a home, work or other phone number)

1. This is the best number [ ]
2. Home number __________
3. Work number __________
4. Mobile number __________
5. Other number __________

Sequence guide: If A.10=1 & A.11 = 2, go to A.23.
If A.11A = 1 Go to A.23.
If A.13=1 Go to A.23.
Else Go to B.

A.22 (if participant has died) **<u>Their</u> past involvement in the study has been most valuable in providing information that is helping to improve the health of the community. Thank you for your time today.**

1. Cause of death (if volunteered) ________
2. Date of death (if volunteered) ________
3. No details give / unknown [ ]

END/TERMINATION OF INTERVIEW.

A.23 Your involvement in the study has been most valuable in providing information that is helping to improve the health of the community. Thank you for your time today.

END/TERMINATION OF INTERVIEW.

A.24 Record forwarding address and/or telephone number for study participant.

1. Forwarding address __________________
2. Forwarding telephone number __________
3. No details give / unknown [ ]

B. HEALTH CONDITIONS

B.1 I would like to ask you a few questions about your health. We have asked some of these questions before, but we need to ask them again to update our information. Have you ever been told by a doctor that you have any of the following conditions?

(Read options. Multiple response)

1. **Heart attack** [ ]
2. **Stroke** [ ]
3. **Angina** [ ]
4. **Transient Ischaemic Attack/ mini-stroke** [ ]
5. **Osteoporosis** [ ]
6. None [ ]
7. Don't know / refused [ ]

B.2 Have you ever been told by a doctor that you have arthritis? If yes, prompt **what type?**

(Read options if necessary. Multiple response)

1. **Osteoarthritis** [ ]
2. **Rheumatoid arthritis** [ ]
3. **Yes, other** (specify) [ ]
4. Yes, don't know type [ ]
5. No, don't have arthritis [ ]
6. Don't know / refused [ ]

C. HEALTH CARE UTILISATION

C.1 How many times in the last 12 months have you used these health services in South Australia?

(Multiple Response. Read options. *Interviewer note: enter number of times for each service used. If unsure, approximate number will do.*)

1. **General practitioner** ____
2. **Community health centre** ____
3. **District nurses or other community nurses** ____
4. **Psychologist** ____
5. **Psychiatrist** ____
6. **Day surgery** ____
7. **Hospital – Accident & Emergency Department** ____
8. **Hospital – Clinic (outpatient/ specialist/allied health)** ____
9. **Eye specialist/ ophthalmologist** ____
10. **Other specialist doctor (not in a hospital)** ____
11. **Physiotherapist** ____
12. **Chiropractor** ____
13. **Alternative therapist (eg. naturopath, osteopath)** ____
14. **Podiatrist** ____
15. **Dietician** ____
16. **Nurse educator** ____
17. **Social Worker / Counsellor** ____
18. **Other** (please specify) ____

D. LOW BACK

These next few questions are about your low back.

D.1 Have you ever had pain or aching in your low back, either at rest or when moving, on <u>most days</u> for at <u>least a month</u>?

(Single response)

1. Yes [ ]
2. No [ ]
3. Don't know / refused [ ]

D.2 Have you ever had stiffness in your low back, when first getting out of bed in the morning, <u>on most days</u> for at <u>least a month</u>?

(Single response)

1. Yes [ ]
2. No [ ]
3. Don't know / refused [ ]

Sequence guide: If D.2 = 2 or 3 go to E.

D.3 [Did / does] this stiffness last at least 15 minutes?

(Single response)

1. Yes [ ]
2. No [ ]
3. Don't know / refused [ ]

E. HIPS

These next few questions are about your hips.

E.1 Thinking of your hips, have you ever had a serious joint strain or sprain lasting two or more weeks, a fracture or a dislocation?

(Single response. Interviewer note: hip includes buttock, groin and side of upper thigh)

1. Yes [ ]
2. No [ ]
3. Don't know [ ]

Sequence guide: If E.1= 2 or 3, go to E.3.

E.2 Have you had your hip condition treated with an operation?

(Single response. Interviewer note: hip includes buttock, groin and side of upper thigh)

1. Yes [ ]
2. No [ ]
3. Don't know [ ]

E.3 Have you had a hip joint replacement?

(Single response. Interviewer prompt which hip/s if yes)

1. Yes - left hip [ ]
2. Yes - right hip [ ]
3. Yes - both hips [ ]
4. No [ ]
5. Don't know / refused [ ]

Sequence guide: If E.3 = 3, go to F.

E.4 Have you ever had pain or aching in your hips, either at rest or when moving, on <u>most days</u> for at <u>least a month</u>?

(Single response. Interviewer note: hip includes buttock, groin and side of upper thigh)

1. Yes [ ]
2. No [ ]
3. Don't know / refused [ ]

E.5 Have you ever had stiffness in your hip joints or muscles, when first getting out of bed in the morning, on most days for at least a month?

(Single response. Interviewer note: hip includes buttock, groin and side of upper thigh)

1. Yes [ ]
2. No [ ]
3. Don't know / refused [ ]

Sequence guide:

If E.4 = 2 or 3 AND E.5 = 2 or 3, go to F.

If E.5 = 2 or 3, go to E.7

E.6 [Did / Does] this stiffness last at least 15 minutes?

(Single response)

1. Yes [ ]
2. No [ ]
3. Don't know / refused [ ]

E.7 [Was / Is] your hip pain, aching or stiffness only as a result of a sprain, strain, fracture or dislocation?

(Single response. Interviewer note: hip includes buttock, groin and side of upper thigh)

1. Yes [ ]
2. No [ ]
3. Don't know / refused [ ]

F. KNEES

The next questions are about your knees, including the back of your knee.

F.1 Thinking of your knees, have you ever had a serious joint strain or sprain lasting two or more weeks, cartilage or meniscus damage, a fracture or a dislocation?

(Single response)

1. Yes [ ]
2. No [ ]
3. Don't know [ ]

Sequence guide: If F.1 = 2 or 3, go to F.3.

F.2 Have you had your strain, sprain, cartilage or meniscus damage, fracture or dislocation, treated with an operation?

(Single response)

1. Yes [ ]
2. No [ ]
3. Don't know [ ]

F.3 Have you had a knee joint replacement?

(Single response. Interviewer prompt which hip/s if yes)

1. Yes – one knee [ ]
2. Yes - both knees [ ]
3. No [ ]
4. Don't know / refused [ ]

Sequence guide: If F.3 = 2, go to G.

F.4 Have you ever had pain, aching or stiffness in your knees, either at rest or when moving, on most days for at least a month?

(Single response. Interviewer note: includes back of knee.)

1. Yes [ ]
2. No [ ]
3. Don't know / refused [ ]

Sequence guide: If F.4= 2 or 3, go to G.

F.5 [Was/Is] your knee pain, aching or stiffness only as a result of a sprain, strain, cartilage or meniscus damage, fracture or dislocation?

(Single response)

1. Yes [ ]
2. No [ ]
3. Don't know / refused [ ]

(interviewer note: if both knees are involved, then questions relate to the worst knee)

WOMAC™ OSTEOARTHRITIS INDEX

© Professor Nicholas Bellamy

(Interviewer note: See briefing notes re questions if necessary. If respondent says that they don't do, or try to avoid an activity, prompt to respond if they hypothetically had to do it)

Think about the pain you felt in your knee during the last 2 weeks.

F.6 How much pain do you have … WALKING ON A FLAT SURFACE?

(Read options. Single response. Interviewer note: refers to walking on even surface eg sidewalk or in shops)

1. **None** [ ]
2. **Mild** [ ]
3. **Moderate** [ ]
4. **Severe** [ ]
5. **Extreme** [ ]
6. Don't know / refused [ ]
7. I have had no pain at all with <u>any</u> activity in the last two weeks [ ]

If F.6 = 7. Go to F.11

F.7 [Think about the pain you felt in your knee during the last 2 weeks, how much pain do you have] … GOING UP OR DOWN STAIRS?

(Read options if necessary. Single response. Interviewer note: If one way worse than other rate according to most extreme pain. If respondent avoids answering, ask how much pain if they hypothetically had to do it.)

1. **None** [ ]
2. **Mild** [ ]
3. **Moderate** [ ]
4. **Severe** [ ]
5. **Extreme** [ ]
6. Don't know / refused [ ]

F.8 [Think about the pain you felt in your knee during the last 2 weeks, how much pain do you have] … AT NIGHT WHILE IN BED, ie pain that disturbs your sleep?

(Read options if necessary. Single response. Interviewer note: refers to pain that disturbs sleep)

1. **None** [ ]
2. **Mild** [ ]
3. **Moderate** [ ]
4. **Severe** [ ]
5. **Extreme** [ ]
6. Don't know / refused [ ]

F.9 [Think about the pain you felt in your knee during the last 2 weeks, how much pain do you have] … SITTING OR LYING?

(Read options if necessary. Single response. Interviewer note: pain while sitting in chair or lying awake in bed)

1. **None** [ ]
2. **Mild** [ ]
3. **Moderate** [ ]
4. **Severe** [ ]
5. **Extreme** [ ]
6. Don't know / refused [ ]

F.10 [Think about the pain you felt in your knee during the last 2 weeks, how much pain do you have] … STANDING UPRIGHT?

(Read options if necessary. Single response. Interviewer note: if get pain while in standing position but not moving)

1. **None** [ ]
2. **Mild** [ ]
3. **Moderate** [ ]
4. **Severe** [ ]
5. **Extreme** [ ]
6. Don't know / refused [ ]

© Professor Nicholas Bellamy

F.11 Think about the stiffness (not pain) you felt in your knee during the last 2 weeks. Stiffness is a sensation of decrease ease in moving your joint.
How severe is your stiffness AFTER FIRST AWAKENING in the morning?

(Read options if necessary. Single response)

1. **None** [ ]
2. **Mild** [ ]
3. **Moderate** [ ]
4. **Severe** [ ]
5. **Extreme** [ ]
6. Don't know / refused [ ]
7. I have had no stiffness at all with any activity in the past two weeks [ ]

If F.11 = 7. Go to F.13

F.12 How severe is your stiffness after sitting, lying or resting LATER IN THE DAY?

(Read options if necessary. Single response)

1. **None** [ ]
2. **Mild** [ ]
3. **Moderate** [ ]
4. **Severe** [ ]
5. **Extreme** [ ]
6. Don't know / refused [ ]

F.13 Think about the difficulty you had in doing the following daily physical activities due to your knee during the last 2 weeks. By this, we mean your ability to move around and to look after yourself.

[Thinking about your knees in the last 2 weeks, what degree of difficulty do you have] … DESCENDING STAIRS?

(Read options if necessary. Single response. If respondent avoids, then asked how much pain if they hypothetically had to do it.)

1. **None** [ ]
2. **Mild** [ ]
3. **Moderate** [ ]
4. **Severe** [ ]
5. **Extreme** [ ]
6. Don't know / refused [ ]

F.14 [Thinking about your knees in the last 2 weeks, what degree of difficulty do you have] … **ASCENDING STAIRS?**

(Read options if necessary. Single response. If respondent avoids, then asked how much pain if they hypothetically had to do it.)

1. **None** [ ]
2. **Mild** [ ]
3. **Moderate** [ ]
4. **Severe** [ ]
5. **Extreme** [ ]
6. Don't know / refused [ ]

F.15 [Thinking about your knees in the last 2 weeks, what degree of difficulty do you have] … **RISING FROM SITTING?**

(Read options if necessary. Single response.)

1. **None** [ ]
2. **Mild** [ ]
3. **Moderate** [ ]
4. **Severe** [ ]
5. **Extreme** [ ]
6. Don't know / refused [ ]

F.16 [Thinking about your knees in the last 2 weeks, what degree of difficulty do you have] … **STANDING?**

(Read options if necessary. Single response. Interviewer note: Degree of difficulty remaining in a standing position)

1. **None** [ ]
2. **Mild** [ ]
3. **Moderate** [ ]
4. **Severe** [ ]
5. **Extreme** [ ]
6. Don't know / refused [ ]

F.17 [Thinking about your knees in the last 2 weeks, what degree of difficulty do you have] … **BENDING TO THE FLOOR?**

(Read options if necessary. Single response. Interviewer note: Can bend down using back or squat)

1. **None** [ ]
2. **Mild** [ ]
3. **Moderate** [ ]
4. **Severe** [ ]
5. **Extreme** [ ]
6. Don't know / refused [ ]

© Professor Nicholas Bellamy

F.18 [Thinking about your knees in the last 2 weeks, what degree of difficulty do you have] … **WALKING ON *A* FLAT SURFACE?**

(Read options if necessary. Single response. Interviewer note: walk on even surface eg sidewalk or in shops)

1. **None** [ ]
2. **Mild** [ ]
3. **Moderate** [ ]
4. **Severe** [ ]
5. **Extreme** [ ]
6. Don't know / refused [ ]

F.19 [Thinking about your knees in the last 2 weeks, what degree of difficulty do you have] … **GETTING IN OR OUT OF THE CAR, OR GETTING ON OR OFF A BUS?**

(Read options if necessary. Single response. Interviewer note: Can be either driver or passenger, if degree differs between getting in or out, or on or off bus, rate direction which produces greatest difficulty)

1. **None** [ ]
2. **Mild** [ ]
3. **Moderate** [ ]
4. **Severe** [ ]
5. **Extreme** [ ]
6. Don't know / refused [ ]

F.20 [Thinking about your knees in the last 2 weeks, what degree of difficulty do you have] … **GOING SHOPPING?**

(Read options if necessary. Single response)

1. **None** [ ]
2. **Mild** [ ]
3. **Moderate** [ ]
4. **Severe** [ ]
5. **Extreme** [ ]
6. Don't know / refused [ ]

F.21 [Thinking about your knees in the last 2 weeks, what degree of difficulty do you have] … **PUTTING ON YOUR SOCKS OR STOCKINGS?**

(Read options if necessary. Single response. If respondent avoids, then asked how much pain if they hypothetically had to do it.)

1. **None** [ ]
2. **Mild** [ ]
3. **Moderate** [ ]
4. **Severe** [ ]
5. **Extreme** [ ]
6. Don't know / refused [ ]

F.22 [Thinking about your knees in the last 2 weeks, what degree of difficulty do you have] … **RISING FROM BED?**

(Read options if necessary. Single response

Interviewer note: act of swinging legs over side of bed and standing up)

1. **None** [ ]
2. **Mild** [ ]
3. **Moderate** [ ]
4. **Severe** [ ]
5. **Extreme** [ ]
6. Don't know / refused [ ]

F.23 [Thinking about your knees in the last 2 weeks, what degree of difficulty do you have] … **TAKING OFF YOUR SOCKS OR STOCKINGS?**

(Read options if necessary. Single response)

1. **None** [ ]
2. **Mild** [ ]
3. **Moderate** [ ]
4. **Severe** [ ]
5. **Extreme** [ ]
6. Don't know / refused [ ]

© Professor Nicholas Bellamy

F.24 [Thinking about your knees in the last 2 weeks, what degree of difficulty do you have] … **LYING IN BED?**

(Read options if necessary. Single response

Interviewer note: difficulty turning from side to side or staying in one position in lying posture)

1. **None** [ ]
2. **Mild** [ ]
3. **Moderate** [ ]
4. **Severe** [ ]
5. **Extreme** [ ]
6. Don't know / refused [ ]

F.25 [Thinking about your knees in the last 2 weeks, what degree of difficulty do you have] … **GETTING IN OR OUT OF THE BATH?**

(Read options if necessary. Single response Interviewer note: If only shower, record degree of difficulty getting in and out of shower. If difficulty different between getting in and out, rate activity which produces greatest difficulty)

1. **None** [ ]
2. **Mild** [ ]
3. **Moderate** [ ]
4. **Severe** [ ]
5. **Extreme** [ ]
6. Don't know / refused [ ]

F.26 [Thinking about your knees in the last 2 weeks, what degree of difficulty do you have] … **SITTING?**

(Read options if necessary. Single response Interviewer note: sitting in chair for a prolonged period)

1. **None** [ ]
2. **Mild** [ ]
3. **Moderate** [ ]
4. **Severe** [ ]
5. **Extreme** [ ]
6. Don't know / refused [ ]

F.27 [Thinking about your knees in the last 2 weeks, what degree of difficulty do you have] … GETTING ON OR OFF THE TOILET?

(Read options if necessary. Single response Interviewer note: If degree of difficulty different for two actions rate action that is the most difficult)

1. **None** [ ]
2. **Mild** [ ]
3. **Moderate** [ ]
4. **Severe** [ ]
5. **Extreme** [ ]
6. Don't know / refused [ ]

F.28 [Thinking about your knees in the last 2 weeks, what degree of difficulty do you have] … (**with) HEAVY DOMESTIC DUTIES?**

(Read options if necessary. Single response

Interviewer note: Activities such as mowing, shovelling, vacuuming, moving heavy objects)

1. **None** [ ]
2. **Mild** [ ]
3. **Moderate** [ ]
4. **Severe** [ ]
5. **Extreme** [ ]
6. Don't know / refused [ ]

F.29 [Thinking about your knees in the last 2 weeks, what degree of difficulty do you have] … (**with) LIGHT DOMESTIC DUTIES?**

(Read options if necessary. Single response

Interviewer note: activities include crafts, hobbies, clearing table, cooking, dusting)

1. **None** [ ]
2. **Mild** [ ]
3. **Moderate** [ ]
4. **Severe** [ ]
5. **Extreme** [ ]
6. Don't know / refused [ ]

© Professor Nicholas Bellamy

F.30 Have you ever been told by a doctor that you have arthritis in your knee? if yes, prompt **which type?**

(Read options if necessary. Multiple response)

1. Yes - osteoarthritis [ ]
2. Yes - rheumatoid arthritis [ ]
3. Yes – don't know type [ ]
4. No [ ]
5. Don't know [ ]

G. FEET

Now I'm going to ask some questions about your feet.

G.1 On most days, do you have pain, aching or stiffness in either of your feet?

(Single response. Interviewer note: prompt which foot if necessary)

1. No [ ]
2. Yes, left foot [ ]
3. Yes, right foot [ ]
4. Yes, both feet [ ]
5. Yes, not sure what side [ ]
6. Not applicable (eg amputee) [ ]
7. Don't Know [ ]

Sequence guide: If G.1 = 1, go to H.

If G.1 = 3, go to G.4

If G.1 = 5, go to G.6

If G.1 = 6, 7 go to H.

G.2 Thinking of your left foot, is the pain, aching or stiffness … mild, moderate or severe?

(Single response)

1. Mild [ ]
2. Moderate [ ]
3. Severe [ ]
4. Don't Know [ ]

G.3 Still thinking about your left foot, how long have you had this pain, aching or stiffness?

(Single response, read options)

1. **Less than 6 months** [ ]
2. **6 months to less than 1 year** [ ]
3. **1 year to less than 5 years** [ ]
4. **5 years to less than 10 years** [ ]
5. **10 years or more** [ ]
6. Don't know [ ]

Sequence guide: If G.1 = 2, go to H.

G.4 Thinking of your right foot, is the pain, aching or stiffness … mild, moderate or severe?

(Single response)

1. Mild [ ]
2. Moderate [ ]
3. Severe [ ]
4. Don't know [ ]

G.5 Still thinking about your right foot, how long have you had this pain, aching or stiffness?

(Single response, read options)

1. **Less than 6 months** [ ]
2. **6 months to less than 1 year** [ ]
3. **1 year to less than 5 years** [ ]
4. **5 years to less than 10 years** [ ]
5. **10 years or more** [ ]
6. Don't know [ ]

Sequence guide: If G.1 = 3 go to H.

If G.1 = 4 go to H.

G.6 Is the pain, aching or stiffness … mild, moderate or severe?

(Single response)

1. Mild [ ]
2. Moderate [ ]
3. Severe [ ]
4. Don't know [ ]

G.7 How long have you had this pain, aching or stiffness?

(Single response, read options)

1. **Less than 6 months** [ ]
2. **6 months to less than 1 year** [ ]
3. **1 year to less than 5 years** [ ]
4. **5 years to less than 10 years** [ ]
5. **10 years or more** [ ]
6. Don't know [ ]

H. SHOULDER PAIN OR STIFFNESS

The next few questions are about shoulder pain and stiffness. Shoulder pain and stiffness includes the shoulder joint, top of shoulder, shoulder blade and upper arm, not your neck.

H.1 Have you ever had pain or aching in your shoulder, either at rest or when moving, <u>on most days for at least a month</u>?

(Single response)

1. Yes [ ]
2. No [ ]
3. Don't know / refused [ ]

H.2 Have you ever had stiffness in your shoulder, when first getting out of bed in the morning, on most days for at least a month?

(Single response)

1. Yes [ ]
2. No [ ]
3. Don't know / refused [ ]

Sequence guide:

If H.1 = 1 and H.2 = 2 or 3, go to H.4.

If H.1 = 2 or 3 <u>and</u> H.2 = 2 or 3, go to I.

H.3 [Did / Does] this stiffness last at least 15 minutes?

(Single response)

1. Yes [ ]
2. No [ ]
3. Don't know / refused [ ]

If H.1 = 2 or 3 and H.2 = 1, go to H.9.

Shoulder Pain and Disability Index

(Interviewer note: if both shoulders are involved, the questions relate to the worst shoulder. If respondent says don't do, or try to avoid, an activity prompt to respond if they hypothetically had to do it)

These next questions relate to your shoulder area.

H.4 [Thinking about THE LAST WEEK, please describe your pain on a scale from 0 to 10, where 0 is no pain and 10 is the worst pain imaginable. How severe is your pain] … AT ITS WORST?

(Single response. Interviewer note: repeat scale if necessary)

0. No pain at all [ ]
1. 1 [ ]
2. 2 [ ]
3. 3 [ ]
4. 4 [ ]
5. 5 [ ]
6. 6 [ ]
7. 7 [ ]
8. 8 [ ]
9. 9 [ ]
10. The worst pain imaginable [ ]
11. Don't know [ ]
12. No pain in shoulder in the last week [ ]

Sequence guide: If H.4 = 12 Go to H.9

H.5 [Thinking about your shoulder in the last week, how severe is your pain … on a scale from 0 to 10, where 0 is no pain and 10 is the worst pain imaginable] … WHEN LYING ON THE INVOLVED SIDE?

(Single response. Interviewer note: repeat scale if necessary)

0. No pain at all [ ]
1. 1 [ ]
2. 2 [ ]
3. 3 [ ]
4. 4 [ ]
5. 5 [ ]
6. 6 [ ]
7. 7 [ ]
8. 8 [ ]
9. 9 [ ]
10. The worst pain imaginable [ ]
11. Don't know

H.6 [Thinking about your shoulder in the last week, how severe is your pain … on a scale from 0 to 10, where 0 is no pain and 10 is the worst pain imaginable] … REACHING FOR SOMETHING ON A HIGH SHELF?

(Single response. Interviewer note: repeat scale if necessary. If respondent avoids, then asked how much pain if they hypothetically had to do it.)

0. No pain at all [ ]
1. 1 [ ]
2. 2 [ ]
3. 3 [ ]
4. 4 [ ]
5. 5 [ ]
6. 6 [ ]
7. 7 [ ]
8. 8 [ ]
9. 9 [ ]
10. The worst pain imaginable [ ]
11. Don't know

H.7 [Thinking about your shoulder in the last week, how severe is your pain … on a scale from 0 to 10, where 0 is no pain and 10 is the worst pain imaginable] … TOUCHING THE BACK OF YOUR NECK?

(Single response. Interviewer note: repeat scale if necessary)

0. No pain at all [ ]
1. 1 [ ]
2. 2 [ ]
3. 3 [ ]
4. 4 [ ]
5. 5 [ ]
6. 6 [ ]
7. 7 [ ]
8. 8 [ ]
9. 9 [ ]
10. The worst pain imaginable [ ]
11. Don't know

H.8 [Thinking about your shoulder in the last week, how severe is your pain … on a scale from 0 to 10, where 0 is no pain and 10 is the worst pain imaginable] … PUSHING WITH THE INVOLVED ARM?

(Single response. Interviewer note: repeat scale if necessary. If respondent avoids, then asked how much pain if they hypothetically had to do it.)

0. No pain at all [ ]
1. 1 [ ]
2. 2 [ ]
3. 3 [ ]
4. 4 [ ]
5. 5 [ ]
6. 6 [ ]
7. 7 [ ]
8. 8 [ ]
9. 9 [ ]
10. The worst pain imaginable [ ]
11. Don't know

H.9 For these next questions, can you please describe your experience on a scale from 0 to 10, where 0 is no difficulty and 10 is so difficult it requires help. So thinking about your shoulder area in the last week, how much difficulty do you have …WASHING YOUR HAIR?

(Single response. Interviewer note: repeat scale if necessary)

0. No difficulty [ ]
1. 1 [ ]
2. 2 [ ]
3. 3 [ ]
4. 4 [ ]
5. 5 [ ]
6. 6 [ ]
7. 7 [ ]
8. 8 [ ]
9. 9 [ ]
10. So difficult it requires help [ ]
11. Don't know

H.10 [Thinking about your shoulder in the last week, how much difficulty do you have … on a scale from 0 to 10, where 0 is no difficulty and 10 is so difficult it requires help] … WASHING YOUR BACK?

(Single response. Interviewer note: repeat scale if necessary)

0. No difficulty [ ]
1. 1 [ ]
2. 2 [ ]
3. 3 [ ]
4. 4 [ ]
5. 5 [ ]
6. 6 [ ]
7. 7 [ ]
8. 8 [ ]
9. 9 [ ]
10. So difficult it requires help [ ]
11. Don't know

H.11 [Thinking about your shoulder in the last week, how much difficulty do you have … on a scale from 0 to 10, where 0 is no difficulty and 10 is so difficult it requires help]… PUTTING ON AN UNDERSHIRT OR JUMPER?

(Single response. Interviewer note: repeat scale if necessary)

0. No difficulty [ ]
1. 1 [ ]
2. 2 [ ]
3. 3 [ ]
4. 4 [ ]
5. 5 [ ]
6. 6 [ ]
7. 7 [ ]
8. 8 [ ]
9. 9 [ ]
10. So difficult it requires help [ ]
11. Don't know

H.12 [Thinking about your shoulder in the last week, how much difficulty do you have … on a scale from 0 to 10, where 0 is no difficulty and 10 is so difficult it requires help]…PUTTING ON A SHIRT THAT BUTTONS DOWN THE FRONT?

(Single response. Interviewer note: repeat scale if necessary)

0. No difficulty [ ]
1. 1 [ ]
2. 2 [ ]
3. 3 [ ]
4. 4 [ ]
5. 5 [ ]
6. 6 [ ]
7. 7 [ ]
8. 8 [ ]
9. 9 [ ]
10. So difficult it requires help [ ]
11. Don't know

H.13 [Thinking about your shoulder in the last week, how much difficulty do you have … on a scale from 0 to 10, where 0 is no difficulty and 10 is so difficult it requires help] … PUTTING ON YOUR PANTS?

(Single response. Interviewer note: repeat scale if necessary)

0. No difficulty [ ]
1. 1 [ ]
2. 2 [ ]
3. 3 [ ]
4. 4 [ ]
5. 5 [ ]
6. 6 [ ]
7. 7 [ ]
8. 8 [ ]
9. 9 [ ]
10. So difficult it requires help [ ]
11. Don't know

H.14 [Thinking about your shoulder in the last week, how much difficulty do you have … on a scale from 0 to 10, where 0 is no difficulty and 10 is so difficult it requires help]… PLACING AN OBJECT ON A HIGH SHELF?

(Single response. Interviewer note: repeat scale if necessary)

0. No difficulty [ ]
1. 1 [ ]
2. 2 [ ]
3. 3 [ ]
4. 4 [ ]
5. 5 [ ]
6. 6 [ ]
7. 7 [ ]
8. 8 [ ]
9. 9 [ ]
10. So difficult it requires help [ ]
11. Don't know

H.15 [Thinking about your shoulder in the last week, how much difficulty do you have … on a scale from 0 to 10, where 0 is no difficulty and 10 is so difficult it requires help] … CARRYING A HEAVY OBJECT OF 10 POUNDS (4.5 kilograms)?

(Single response. Interviewer note: repeat scale if necessary. Suggest large bag of potatoes if participant has difficulty with quantity)

0. No difficulty [ ]
1. 1 [ ]
2. 2 [ ]
3. 3 [ ]
4. 4 [ ]
5. 5 [ ]
6. 6 [ ]
7. 7 [ ]
8. 8 [ ]
9. 9 [ ]
10. So difficult it requires help [ ]
11. Don't know

H.16 [Thinking about your shoulder in the last week, how much difficulty do you have … on a scale from 0 to 10, where 0 is no difficulty and 10 is so difficult it requires help]… REMOVING SOMETHING FROM YOUR BACK POCKET?

(Single response. Interviewer note: repeat scale if necessary. For female participants who may not relate to this activity, suggest reaching around to the lower back)

0. No difficulty [ ]
1. 1 [ ]
2. 2 [ ]
3. 3 [ ]
4. 4 [ ]
5. 5 [ ]
6. 6 [ ]
7. 7 [ ]
8. 8 [ ]
9. 9 [ ]
10. So difficult it requires help [ ]
11. Don't know

I. HAND PAIN OR STIFFNESS

The next questions are about your hands.

I.1 Have you had pain, aching or stiffness in your hands, either at rest or when using them, <u>on most days for at least a month</u>?

(Single response)

1. Yes [ ]
2. No [ ]
3. Don't know / refused [ ]

Sequence guide: If I.1= 2 or 3, go to J.

AUSCAN© Osteoarthritis Hand Index

© Professor Nicholas Bellamy

(Interviewer note: See briefing notes re questions if necessary. If respondent says they don't do, or try to avoid, an activity prompt to respond if they hypothetically had to do it)

Think about the pain you felt in your hands during the last 48 hours, how much pain do you have in your hands …

I.2 …**AT REST (ie when <u>not</u> using your hands)?**

(Read options if necessary. Single response)

1. **None** [ ]
2. **Mild** [ ]
3. **Moderate** [ ]
4. **Severe** [ ]
5. **Extreme** [ ]
6. Don't know / refused [ ]
7. No pain at all with any activity over last 48 hours [ ]

If I.2 = 7 Go to I.7

I.3 [Think about the pain you felt in your hands during the last 48 hours, how much pain do you have in your hands] … **WHEN GRIPPING OBJECTS WITH YOUR HANDS?**

(Read options if necessary. Single response)

1. **None** [ ]
2. **Mild** [ ]
3. **Moderate** [ ]
4. **Severe** [ ]
5. **Extreme** [ ]
6. Don't know / refused [ ]

I.4 [Think about the pain you felt in your hands during the last 48 hours, how much pain do you have in your hands] … **WHEN LIFTING OBJECTS WITH YOUR HANDS?**

(Read options if necessary. Single response)

1. **None** [ ]
2. **Mild** [ ]
3. **Moderate** [ ]
4. **Severe** [ ]
5. **Extreme** [ ]
6. Don't know / refused [ ]

I.5 [Think about the pain you felt in your hands during the last 48 hours, how much pain do you have in your hands] … **WHEN TURNING OBJECTS WITH YOUR HANDS?**

(Read options if necessary. Single response Interviewer note: rotating objects like door handles, if they have special handles, pain when turning normal handles)

1. **None** [ ]
2. **Mild** [ ]
3. **Moderate** [ ]
4. **Severe** [ ]
5. **Extreme** [ ]
6. Don't know / refused [ ]

I.6 [Think about the pain you felt in your hands during the last 48 hours, how much pain do you have in your hands] … **WHEN SQUEEZING OBJECTS WITH YOUR HANDS?**

(Read options if necessary. Single response)

1. **None** [ ]
2. **Mild** [ ]
3. **Moderate** [ ]
4. **Severe** [ ]
5. **Extreme** [ ]
6. Don't know / refused [ ]

© Professor Nicholas Bellamy

Think about the stiffness (not pain) you felt in your hands during the last 48 hours. Stiffness is a sensation of <u>decreased</u> ease with which you move your hands.

I.7 How severe is stiffness in your hands AFTER FIRST WAKENING in the morning?

(Read options if necessary. Single response)

1. **None** [ ]
2. **Mild** [ ]
3. **Moderate** [ ]
4. **Severe** [ ]
5. **Extreme** [ ]
6. Don't know / refused [ ]

Think about the difficulty you had in doing the following daily physical activities during the last 48 hours. By this, we mean <u>your ability to move around and to look after yourself.</u>

I.8 What degree of difficulty do you have] … TURNING TAPS/FAUCETS ON?

(Read options if necessary. Single response Interviewer note: difficulty when turning normal taps, if they have special taps difficulty with normal taps)

1. **None** [ ]
2. **Mild** [ ]
3. **Moderate** [ ]
4. **Severe** [ ]
5. **Extreme** [ ]
6. Don't know / refused [ ]

I.9 [Thinking about your hands, in the last 48 hours, what degree of difficulty do you have] … TURNING A ROUND DOORKNOB OR HANDLE?

(Read options if necessary. Single response Interviewer note: difficulty when turning door handles, if they have special door handles, difficulty with normal handles)

1. **None** [ ]
2. **Mild** [ ]
3. **Moderate** [ ]
4. **Severe** [ ]
5. **Extreme** [ ]
6. Don't know / refused [ ]

I.10 [Thinking about your hands, in the last 48 hours, what degree of difficulty do you have] … DOING UP BUTTONS?

(Read options if necessary. Single response)

1. **None** [ ]
2. **Mild** [ ]
3. **Moderate** [ ]
4. **Severe** [ ]
5. **Extreme** [ ]
6. Don't know / refused [ ]

I.11 [Thinking about your hands, in the last 48 hours, what degree of difficulty do you have] … FASTENING JEWELLERY (ie watches, earrings, cufflinks, necklaces, brooches and bracelets)?

(Read options if necessary. Single response)

1. **None** [ ]
2. **Mild** [ ]
3. **Moderate** [ ]
4. **Severe** [ ]
5. **Extreme** [ ]
6. Don't know / refused [ ]

I.12 [Thinking about your hands, in the last 48 hours, what degree of difficulty do you have] … OPENING A NEW JAR?

(Read options if necessary. Single response)

1. **None** [ ]
2. **Mild** [ ]
3. **Moderate** [ ]
4. **Severe** [ ]
5. **Extreme** [ ]
6. Don't know / refused [ ]

I.13 [Thinking about your hands, in the last 48 hours, what degree of difficulty do you have] … CARRYING A FULL POT WITH ONE HAND?

(Read options if necessary. Single response)

1. **None** [ ]
2. **Mild** [ ]
3. **Moderate** [ ]
4. **Severe** [ ]
5. **Extreme** [ ]
6. Don't know / refused [ ]

© Professor Nicholas Bellamy

I.14 [Thinking about your hands, in the last 48 hours, what degree of difficulty do you have] … PEELING VEGETABLES/ FRUIT?

(Read options if necessary. Single response)

1. **None** [ ]
2. **Mild** [ ]
3. **Moderate** [ ]
4. **Severe** [ ]
5. **Extreme** [ ]
6. Don't know / refused [ ]

I.15 [Thinking about your hands, in the last 48 hours, what degree of difficulty do you have] … PICKING UP LARGE HEAVY OBJECTS?

(Read options if necessary. Single response)

1. **None** [ ]
2. **Mild** [ ]
3. **Moderate** [ ]
4. **Severe** [ ]
5. **Extreme** [ ]
6. Don't know / refused [ ]

I.16 [Thinking about your hands, in the last 48 hours, what degree of difficulty do you have] … WRINGING OUT WASHCLOTHS?

(Re options if necessary. Single response)

1. **None** [ ]
2. **Mild** [ ]
3. **Moderate** [ ]
4. **Severe** [ ]
5. **Extreme** [ ]
6. Don't know / refused [ ]

I.17 Have you ever been told by a doctor that you have arthritis in your hands? if yes, prompt **which type?**

(Single response.)

1. Yes - osteoarthritis [ ]
2. Yes - rheumatoid arthritis [ ]
3. Yes – don't know type [ ]
4. No [ ]
5. Don't know [ ]

© Professor Nicholas Bellamy

J. INJURY

J.1 How many falls (including slips, trips and falls to the ground) did you have in the past year?

(Single response)

1. Enter number of falls ____
2. None [ ]
3. Don't know / refused [ ]

Sequence guide: If J.1 = 2 or 3, go to J.3.

J.2 Did you suffer from a fracture as a result of a fall from a standing height or less, in the past year?

(Single response)

1. **Yes** [ ]
2. **No** [ ]
3. **Don't know** [ ]

J.3 Sequence guide: If Male go to K. If Female aged <40, go to K.

Changing the subject now to menopause, because of its relationship to osteoporosis.

J.4 Have you stopped having your periods?

1. Yes [ ]
2. No, but currently going through menopause [ ]
3. No [ ]
4. Don't know [ ]

Sequence guide: If J.4 > 1, go to K.

J.5 How many years since you had your last period?

(Single response)

1. *Enter* number of years ____
2. Don't know / refused [ ]

K. HEALTH CONDITIONS

K.1 In the last 12 months have you been told by a doctor that you have any of the following conditions?

(Read options. Multiple Response)

1. **Anxiety** [ ]
2. **Depression** [ ]
3. **A stress related problem** [ ]
4. **Any other mental health problem** [ ]
5. None [ ]
6. Don't know / refused [ ]

L. CENTRE FOR EPIDEMIOLOGICAL STUDIES DEPRESSION SCALE (CES-D)

The next questions are about how you have been feeling <u>during the past week</u>.

L.1 During the past week, how often were you bothered by things that usually don't bother you?

(Single response, read options)

1. **Rarely or none of the time** (less than 1 day per week) [ ]
2. **Some or a little of the time** (1 to 2 days per week) [ ]
3. **Occasionally or a moderate amount of the time** (3 to 4 days per week) [ ]
4. **Most or all of the time** (5 to 7 days per week) [ ]
5. Don't know [ ]

L.2 [During the past week], how often did you not feel like eating; that is, your appetite was poor?

(Single response, read options)

1. **Rarely or none of the time** (less than 1 day per week) [ ]
2. **Some or a little of the time** (1 to 2 days per week) [ ]
3. **Occasionally or a moderate amount of the time** (3 to 4 days per week) [ ]
4. **Most or all of the time** (5 to 7 days per week) [ ]
5. Don't know [ ]

L.3 [During the past week], how often have you felt that you could not shake off the blues, even with help from your family or friends?

(Single response, read options)

1. **Rarely or none of the time** (less than 1 day per week) [ ]
2. **Some or a little of the time** (1 to 2 days per week) [ ]
3. **Occasionally or a moderate amount of the time** (3 to 4 days per week) [ ]
4. **Most or all of the time** (5 to 7 days per week) [ ]
5. Don't know [ ]

L.4 [During the past week], how often have you felt that you were just as good as other people?

(Single response, read options)

1. **Rarely or none of the time** (less than 1 day per week) [ ]
2. **Some or a little of the time** (1 to 2 days per week) [ ]
3. **Occasionally or a moderate amount of the time** (3 to 4 days per week) [ ]
4. **Most or all of the time** (5 to 7 days per week) [ ]
5. Don't know [ ]

L.5 [During the past week], how often have you had trouble keeping your mind on what you were doing?

(Single response, read options)

1. **Rarely or none of the time** (less than 1 day per week) [ ]
2. **Some or a little of the time** (1 to 2 days per week) [ ]
3. **Occasionally or a moderate amount of the time** (3 to 4 days per week) [ ]
4. **Most or all of the time** (5 to 7 days per week) [ ]
5. Don't know [ ]

L.6 [During the past week], how often have you felt depressed?

(Single response, read options)

1. **Rarely or none of the time** (less than 1 day per week) [ ]
2. **Some or a little of the time** (1 to 2 days per week) [ ]
3. **Occasionally or a moderate amount of the time** (3 to 4 days per week) [ ]
4. **Most or all of the time** (5 to 7 days per week) [ ]
5. Don't know [ ]

L.7 [During the past week], how often have you felt that everything you did was an effort?

(Single response, read options)

1. **Rarely or none of the time** (less than 1 day per week) [ ]
2. **Some or a little of the time** (1 to 2 days per week) [ ]
3. **Occasionally or a moderate amount of the time** (3 to 4 days per week) [ ]
4. **Most or all of the time** (5 to 7 days per week) [ ]
5. Don't know [ ]

L.8 [During the past week], how often have you felt hopeful about the future?

(Single response, read options)

1. **Rarely or none of the time** (less than 1 day per week) [ ]
2. **Some or a little of the time** (1 to 2 days per week) [ ]
3. **Occasionally or a moderate amount of the time** (3 to 4 days per week) [ ]
4. **Most or all of the time** (5 to 7 days per week) [ ]
5. Don't know [ ]

L.9 [During the past week], how often have you thought your life had been a failure?

(Single response, read options)

1. **Rarely or none of the time** (less than 1 day per week) [ ]
2. **Some or a little of the time** (1 to 2 days per week) [ ]
3. **Occasionally or a moderate amount of the time** (3 to 4 days per week) [ ]
4. **Most or all of the time** (5 to 7 days per week) [ ]
5. Don't know [ ]

L.10 [During the past week], how often have you felt fearful?

(Single response, read options)

1. **Rarely or none of the time** (less than 1 day per week) [ ]
2. **Some or a little of the time** (1 to 2 days per week) [ ]
3. **Occasionally or a moderate amount of the time** (3 to 4 days per week) [ ]
4. **Most or all of the time** (5 to 7 days per week) [ ]
5. Don't know [ ]

L.11 [During the past week], how often was your sleep restless?

(Single response, read options)

1. **Rarely or none of the time** (less than 1 day per week) [ ]
2. **Some or a little of the time** (1 to 2 days per week) [ ]
3. **Occasionally or a moderate amount of the time** (3 to 4 days per week) [ ]
4. **Most or all of the time** (5 to 7 days per week) [ ]
5. Don't know [ ]

L.12 [During the past week], how often were you happy?

(Single response, read options)

1. **Rarely or none of the time** (less than 1 day per week) [ ]
2. **Some or a little of the time** (1 to 2 days per week) [ ]
3. **Occasionally or a moderate amount of the time** (3 to 4 days per week) [ ]
4. **Most or all of the time** (5 to 7 days per week) [ ]
5. Don't know [ ]

L.13 [During the past week], how often have you talked less than usual?

(Single response, read options)

1. **Rarely or none of the time** (less than 1 day per week) [ ]
2. **Some or a little of the time** (1 to 2 days per week) [ ]
3. **Occasionally or a moderate amount of the time** (3 to 4 days per week) [ ]
4. **Most or all of the time** (5 to 7 days per week) [ ]
5. Don't know [ ]

L.14 [During the past week], how often have you felt lonely?

(Single response, read options)

1. **Rarely or none of the time** (less than 1 day per week) [ ]
2. **Some or a little of the time** (1 to 2 days per week) [ ]
3. **Occasionally or a moderate amount of the time** (3 to 4 days per week) [ ]
4. **Most or all of the time** (5 to 7 days per week) [ ]
5. Don't know [ ]

L.15 [During the past week], how often were people unfriendly?

(Single response, read options)

1. **Rarely or none of the time** (less than 1 day per week) [ ]
2. **Some or a little of the time** (1 to 2 days per week) [ ]
3. **Occasionally or a moderate amount of the time** (3 to 4 days per week) [ ]
4. **Most or all of the time** (5 to 7 days per week) [ ]
5. Don’t know [ ]

L.16 [During the past week], how often have you enjoyed life?

(Single response, read options)

1. **Rarely or none of the time** (less than 1 day per week) [ ]
2. **Some or a little of the time** (1 to 2 days per week) [ ]
3. **Occasionally or a moderate amount of the time** (3 to 4 days per week) [ ]
4. **Most or all of the time** (5 to 7 days per week) [ ]
5. Don’t know [ ]

L.17 [During the past week], how often have you had crying spells?

(Single response, read options)

1. **Rarely or none of the time** (less than 1 day per week) [ ]
2. **Some or a little of the time** (1 to 2 days per week) [ ]
3. **Occasionally or a moderate amount of the time** (3 to 4 days per week) [ ]
4. **Most or all of the time** (5 to 7 days per week) [ ]
5. Don’t know [ ]

L.18 [During the past week], how often have you felt sad?

(Single response, read options)

1. **Rarely or none of the time** (less than 1 day per week) [ ]
2. **Some or a little of the time** (1 to 2 days per week) [ ]
3. **Occasionally or a moderate amount of the time** (3 to 4 days per week) [ ]
4. **Most or all of the time** (5 to 7 days per week) [ ]
5. Don’t know [ ]

L.19 [During the past week], how often have you felt that people disliked you?

(Single response, read options)

1. **Rarely or none of the time** (less than 1 day per week) [ ]
2. **Some or a little of the time** (1 to 2 days per week) [ ]
3. **Occasionally or a moderate amount of the time** (3 to 4 days per week) [ ]
4. **Most or all of the time** (5 to 7 days per week) [ ]
5. Don’t know [ ]

L.20 [During the past week], how often have you felt you could not get “going”?

(Single response, read options)

1. **Rarely or none of the time** (less than 1 day per week) [ ]
2. **Some or a little of the time** (1 to 2 days per week) [ ]
3. **Occasionally or a moderate amount of the time** (3 to 4 days per week) [ ]
4. **Most or all of the time** (5 to 7 days per week) [ ]
5. Don’t know [ ]

M. TERMINATION OF INTERVIEW

M.1 That concludes the interview. On behalf of the North West Adelaide Health Study, thank you very much for your time.

(Interviewer note any other comments about the interview)

M.2 Date of interview

M.3 Day of week interview undertaken
