# Supplementary material for: Area-level socioeconomic characteristics and incidence of metabolic syndrome: a prospective cohort study
Source: BMC Public Health. 2013 Jul 25;13:681. doi: 10.1186/1471-2458-13-681 (PMC3733986; doi:10.1186/1471-2458-13-681)
Supplement: Additional file 4 — Questionnaire B. NWAH Study 2 - 04/05. [file 1471-2458-13-681-S4.pdf]

Questionnaire B

NWAH Study 2 - 04/05

North West Adelaide Health Study

Parts of this questionnaire may be similar to the first questionnaire you completed for us. As part of the study, we look at changes over time and so ask for your assistance with these topics once again.

Please read the following instructions before answering the questions.

1. Please complete all the questions as per the instructions by placing a tick in the box ☑ that most closely corresponds to your answer.
2. Your answers will remain strictly confidential. Results of the study may be published in a medical journal, but no information that may lead to the identification of any individual will be released.
3. This questionnaire should take approximately 15 minutes to complete.
4. If you have any problems with this questionnaire, please contact:
   Sandy (Clinic Co-ordinator) on ☏ 8222 7866 or
   Janet (Study Co-ordinator) on ☏ 8226 6054.
5. When you have completed the questionnaire, please bring it with you to your clinic appointment at The Queen Elizabeth Hospital or the Lyell McEwin Health Service.

A. GENERAL HEALTH AND WELL BEING

These first questions ask for your views about your health, how you feel and how well you are able to do your usual activities. Please answer each question.

A1 **In general would you say your health is:**

(tick one box only)
- ☐ 1 Excellent
- ☐ 2 Very good
- ☐ 3 Good
- ☐ 4 Fair
- ☐ 5 Poor

A2 **Compared to one year ago, how would you rate your health in general now?**

(tick one box only)
- ☐ 1 Much better now than one year ago
- ☐ 2 Somewhat better now than one year ago
- ☐ 3 About the same as one year ago
- ☐ 4 Somewhat worse now than one year ago
- ☐ 5 Much worse now than one year ago

The next questions relate to activities you might do <u>during a typical day</u>. <u>Please tell us if your health now</u> limits you a lot, limits you a little or does not limit you at all in these activities.

A3 **Vigorous activities, such as running, lifting heavy objects, participating in strenuous sports?**

(tick one box only)
- ☐ 1 Yes, limited a lot
- ☐ 2 Yes, limited a little
- ☐ 3 No, not limited at all

A4 **Moderate activities, such as moving a table, pushing a vacuum cleaner, bowling or playing golf?**

(tick one box only)
- ☐ 1 Yes, limited a lot
- ☐ 2 Yes, limited a little
- ☐ 3 No, not limited at all

A5 **Lifting or carrying groceries?**

(tick one box only)
- ☐ 1 Yes, limited a lot
- ☐ 2 Yes, limited a little
- ☐ 3 No, not limited at all

A6 **Climbing several flights of stairs?**

(tick one box only)
- ☐ 1 Yes, limited a lot
- ☐ 2 Yes, limited a little
- ☐ 3 No, not limited at all **(Go to A8)** →

A7 **Climbing one flight of stairs?**

(tick one box only)
- ☐ 1 Yes, limited a lot
- ☐ 2 Yes, limited a little
- ☐ 3 No, not limited at all

A8 **Bending, kneeling or stooping?**

(tick one box only)
- ☐ 1 Yes, limited a lot
- ☐ 2 Yes, limited a little
- ☐ 3 No, not limited at all

A9 **Walking more than one kilometre?**

(tick one box only)
- ☐ 1 Yes, limited a lot
- ☐ 2 Yes, limited a little
- ☐ 3 No**,** not limited at all **(Go to A12)**

A10 **Walking half a kilometre?**

(tick one box only)
- ☐ 1 Yes, limited a lot
- ☐ 2 Yes, limited a little
- ☐ 3 No, not limited at all **(Go to A12)**

A11 **Walking 100 metres?**

(tick one box only)
- ☐ 1 Yes, limited a lot
- ☐ 2 Yes, limited a little
- ☐ 3 No, not limited at all

A12 **Bathing or dressing yourself?**

(tick one box only)
- ☐ 1 Yes, limited a lot
- ☐ 2 Yes, limited a little
- ☐ 3 No, not limited at all

The following four questions ask you about your physical health and your daily activities.
During the <u>last four weeks</u> have you …

A13 **Had to cut down on the amount of time you spent on work or other activities as a result of your physical health?**

(tick one box only)
- ☐ 1 Yes
- ☐ 2 No

A14 **Accomplished less than you would like as a result of your physical health?**

(tick one box only)
- ☐ 1 Yes
- ☐ 2 No

A15 **Been limited in the kind of work or other activities as a result of your physical health?**

(tick one box only)
- ☐ 1 Yes
- ☐ 2 No

A16 **Had difficulty performing the work or other activities as a result of your physical health (for example, it took extra effort)?**

(tick one box only)
- ☐ 1 Yes
- ☐ 2 No

The following three questions ask you about your emotions and your daily activities.
During the past four weeks have you …

A17 **Had to cut down on the amount of time you spent on work or other activities as a result of any emotional problems such as feeling depressed or anxious?**

(tick one box only)
- ☐ 1 Yes
- ☐ 2 No

A18 **Accomplished less than you would like as a result of any emotional problems?**

(tick one box only)
- ☐ 1 Yes
- ☐ 2 No

A19 **Had to not do work or other activities as carefully as usual as a result of any emotional problems?**

(tick one box only)
- ☐ 1 Yes
- ☐ 2 No

A20 **During the past four weeks, to what extent has your physical health or emotional problems interfered with your normal social activities with family, friends, neighbours or groups? Has it interfered:**

(tick one box only)
- ☐ 1 Not at all
- ☐ 2 Slightly
- ☐ 3 Moderately
- ☐ 4 Quite a bit
- ☐ 5 Extremely

A21 **How much bodily pain have you had during the past four weeks?**

(tick one box only)
- ☐ 1 None **(Go to A23)**
- ☐ 2 Very mild
- ☐ 3 Mild
- ☐ 4 Moderate
- ☐ 5 Severe
- ☐ 6 Very severe

A22 **During the past four weeks, how much did pain interfere with your normal work (including both work outside the home and housework)?**

(tick one box only)
- ☐ 1 Not at all
- ☐ 2 A little bit
- ☐ 3 Moderately
- ☐ 4 Quite a bit
- ☐ 5 Extremely

These questions are about how you feel and how things have been with you during the past four weeks. For each question please give the one answer that comes closest to the way you have been feeling. During the past four weeks….

A23 **Did you feel full of life?**

(tick one box only)
- ☐ 1 All the time
- ☐ 2 Most of the time
- ☐ 3 A good bit of the time
- ☐ 4 Some of the time
- ☐ 5 A little of the time
- ☐ 6 None of the time

A24 **Have you been a very nervous person?**

(tick one box only)
- ☐ 1 All the time
- ☐ 2 Most of the time
- ☐ 3 A good bit of the time
- ☐ 4 Some of the time
- ☐ 5 A little of the time
- ☐ 6 None of the time

A25 **Have you felt so down in the dumps that nothing could cheer you up?**

(tick one box only)
- ☐ 1 All the time
- ☐ 2 Most of the time
- ☐ 3 A good bit of the time
- ☐ 4 Some of the time
- ☐ 5 A little of the time
- ☐ 6 None of the time

A26 **Have you felt calm and peaceful?**

(tick one box only)
- ☐ 1 All the time
- ☐ 2 Most of the time
- ☐ 3 A good bit of the time
- ☐ 4 Some of the time
- ☐ 5 A little of the time
- ☐ 6 None of the time

A27 **Did you have a lot of energy?**

(tick one box only)
- ☐ 1 All the time
- ☐ 2 Most of the time
- ☐ 3 A good bit of the time
- ☐ 4 Some of the time
- ☐ 5 A little of the time
- ☐ 6 None of the time

A28 **Have you felt down?**

(tick one box only)
- ☐ 1 All the time
- ☐ 2 Most of the time
- ☐ 3 A good bit of the time
- ☐ 4 Some of the time
- ☐ 5 A little of the time
- ☐ 6 None of the time

A29 **Did you feel worn out?**

(tick one box only)
- ☐ 1 All the time
- ☐ 2 Most of the time
- ☐ 3 A good bit of the time
- ☐ 4 Some of the time
- ☐ 5 A little of the time
- ☐ 6 None of the time

A30 **Have you been a happy person?**

(tick one box only)

- ☐ 1 All the time
- ☐ 2 Most of the time
- ☐ 3 A good bit of the time
- ☐ 4 Some of the time
- ☐ 5 A little of the time
- ☐ 6 None of the time

A31 **Did you feel tired?**

(tick one box only)

- ☐ 1 All the time
- ☐ 2 Most of the time
- ☐ 3 A good bit of the time
- ☐ 4 Some of the time
- ☐ 5 A little of the time
- ☐ 6 None of the time

A32 **During the past four weeks, how much of the time has your physical health or emotional problems interfered with your social activities (like visiting friends, relatives, etc)?**

(tick one box only)

- ☐ 1 All the time
- ☐ 2 Most of the time
- ☐ 3 A good bit of the time
- ☐ 4 Some of the time
- ☐ 5 A little of the time
- ☐ 6 None of the time

How true or false is each of the following statements for you?

A33 **"I seem to get sick a little easier than other people."**

(tick one box only)

- ☐ 1 Definitely true
- ☐ 2 Mostly true
- ☐ 3 Don't know
- ☐ 4 Mostly false
- ☐ 5 Definitely false

A34 **"I am as healthy as anybody I know."**

(tick one box only)

- ☐ 1 Definitely true
- ☐ 2 Mostly true
- ☐ 3 Don't know
- ☐ 4 Mostly false
- ☐ 5 Definitely false

A35 **"I expect my health to get worse."**

(tick one box only)

- ☐ 1 Definitely true
- ☐ 2 Mostly true
- ☐ 3 Don't know
- ☐ 4 Mostly false
- ☐ 5 Definitely false

A36 **"My health is excellent."**

(tick one box only)

☐ 1 Definitely true
☐ 2 Mostly true
☐ 3 Don't know
☐ 4 Mostly false
☐ 5 Definitely false

B. EXERCISE

The next questions are about exercise you may do for sport, recreation or fitness.

B1 **In the last two weeks, did you do any *walking* for sport, recreation or fitness?**

(tick one box only)

☐ 1 Yes
☐ 2 No **(Go to B4)**

B2 **How many times did you do any *walking* for exercise in the last two weeks?**

1 Enter number of TIMES ________
☐ 99 Don't know

B3 **What was the total amount of time you spent *walking* in the last two weeks?**

1 Enter number of HOURS ________
2 Enter number of MINUTES ________

B4 **In the last 2 weeks, *(apart from walking)* did you do any exercise which caused a moderate increase in your heart rate or breathing?**

(tick one box only)

☐ 1 Yes
☐ 2 No **(Go to B7)**

B5 **How many times did you do any *moderate exercise* in the last two weeks?**

1 Enter number of TIMES ________
☐ 99 Don't know

B6 **What was the total amount of time you spent doing *moderate exercise* in the last two weeks?**

1 Enter number of HOURS ________
2 Enter number of MINUTES ________

B7 **In the last 2 weeks, did you do *any other exercise* which caused a large increase in your heart rate or breathing, that is, *vigorous exercise*?**

(tick one box only)

☐ 1 Yes
☐ 2 No **(Go to C1)**

B8 **How many times did you do any *vigorous exercise* in the last two weeks?**

1 Enter number of TIMES ________
☐ 99 Don't know

B9 **What was the total amount of time you spent doing *vigorous exercise* in the last two weeks?**

1 Enter number of HOURS ________
2 Enter number of MINUTES ________
☐ 99 Don't know

C. FAMILY HISTORY

C1 **Do, or did, any of your relatives have DIABETES? (Blood/first degree relations only)**

(tick all that apply)

- ☐ 1 Mother
- ☐ 2 Father
- ☐ 3 Sister
- ☐ 4 Brother
- ☐ 5 Grandmother
- ☐ 6 Grandfather
- ☐ 7 Other (please specify) ________________
- ☐ 8 No
- ☐ 9 Don't know

C2 **Do, or did, any of your relatives have HEART DISEASE, for example, heart attack or heart failure? (Blood/first degree relations only)**

(tick all that apply)

- ☐ 1 Mother
- ☐ 2 Father
- ☐ 3 Sister
- ☐ 4 Brother
- ☐ 5 Grandmother
- ☐ 6 Grandfather
- ☐ 7 Other (please specify) ________________
- ☐ 8 No
- ☐ 9 Don't know

C3 **Have any of your relatives ever had a STROKE? (Blood/first degree relations only)**

(tick all that apply)

- ☐ 1 Mother
- ☐ 2 Father
- ☐ 3 Sister
- ☐ 4 Brother
- ☐ 5 Grandmother
- ☐ 6 Grandfather
- ☐ 7 Other (please specify) ________________
- ☐ 8 No
- ☐ 9 Don't know

C4 **Do, or did, any of your relatives have OSTEOPOROSIS? (Blood/first degree relations only)**

(tick all that apply)

- ☐ 1 Mother
- ☐ 2 Father
- ☐ 3 Sister
- ☐ 4 Brother
- ☐ 5 Grandmother
- ☐ 6 Grandfather
- ☐ 7 Other (please specify) ________________
- ☐ 8 No
- ☐ 9 Don't know

D. OSTEOPOROSIS

D1 **In the <u>past five years</u>, have you had a FALL, from a standing height or less, that resulted in a fracture of the:**

(tick all that apply)

- ☐ 1 Hip
- ☐ 2 Wrist
- ☐ 3 Upper arm/ shoulder
- ☐ 4 Vertebral bodies (spine)
- ☐ 5 Ribs
- ☐ 6 Ankle
- ☐ 7 Other (please specify) ________________
- ☐ 8 No falls resulting in a fracture
- ☐ 9 No falls

D2 **In the <u>past five years</u>, have you had a MAJOR TRAUMA (eg car accident) that resulted in a fracture of the:**

(tick all that apply)

- ☐ 1 Hip
- ☐ 2 Wrist
- ☐ 3 Upper arm/ shoulder
- ☐ 4 Vertebral bodies (spine)
- ☐ 5 Ribs
- ☐ 6 Ankle
- ☐ 7 Other (please specify) ________________
- ☐ 8 No major traumas resulting in a fracture
- ☐ 9 No major traumas

D3 **In the <u>past five years</u>, have you had a fracture of any of the following due to SOME OTHER KIND OF EVENT - please specify the incident:**

(tick all that apply)

- ☐ 1 Hip
  Incident ______________________________
- ☐ 2 Wrist
  Incident ______________________________
- ☐ 3 Upper arm/ shoulder
  Incident ______________________________
- ☐ 4 Vertebral bodies (spine)
  Incident ______________________________
- ☐ 5 Ribs
  Incident ______________________________
- ☐ 6 Ankle
  Incident ______________________________
- ☐ 7 Other (please specify) ________________
  Incident ______________________________
- ☐ 8 No incidents resulting in a fracture

E. SUNLIGHT

E1 **How much direct sunlight are you exposed to on an average <u>week day</u> during SUMMER?**

(tick one box only)

- ☐ 1 Less than one hour
- ☐ 2 One hour to less than two hours
- ☐ 3 Two hours to less than three hours
- ☐ 4 Three hours to less than four hours
- ☐ 5 Four or more hours

E2 **How much direct sunlight are you exposed to on an average <u>week day</u> during WINTER?**

(tick one box only)

- ☐ 1 Less than one hour
- ☐ 2 One hour to less than two hours
- ☐ 3 Two hours to less than three hours
- ☐ 4 Three hours to less than four hours
- ☐ 5 Four or more hours

E3 **How much direct sunlight are you exposed to on an average <u>weekend</u> day during SUMMER?**

(tick one box only)

- ☐ 1 Less than one hour
- ☐ 2 One hour to less than two hours
- ☐ 3 Two hours to less than three hours
- ☐ 4 Three hours to less than four hours
- ☐ 5 Four or more hours

E4 **How much direct sunlight are you exposed to on an average <u>weekend</u> day during WINTER?**

(tick one box only)

- ☐ 1 Less than one hour
- ☐ 2 One hour to less than two hours
- ☐ 3 Two hours to less than three hours
- ☐ 4 Three hours to less than four hours
- ☐ 5 Four or more hours

E5 **Suppose your skin was exposed to strong sunshine at the beginning of summer with no protection at all. If you stayed in the sun for 30 minutes, would your skin …**

(tick one box only)

- ☐ 1 Always burn never tan
- ☐ 2 Always burn sometimes tan
- ☐ 3 Always burn always tan
- ☐ 4 Sometimes burn always tan
- ☐ 5 Rarely burn always tan
- ☐ 6 Never burn always tan
- ☐ 7 Other (please specify) _______________

F. DIABETES

F1 **Have you ever been told by a doctor that you have diabetes?**

(tick one box only)

☐ 1 Yes **(If MALE, go to F4)** **(If FEMALE, go to F2)**

☐ 2 No **(Go to G1)**

FOR WOMEN WHO SAID YES TO F1

F2 **Were you pregnant when you were first told you had diabetes?**

(tick one box only)

☐ 1 Yes

☐ 2 No

F3 **Have you ever been told that you had diabetes other than when you were pregnant?**

(tick one box only)

☐ 1 Yes

☐ 2 No **(Go to G1)**

F4 **Other than gestational diabetes, what type of diabetes were you told you had?**

(tick one box only)

☐ 1 Type 1 (Insulin dependent or Juvenile onset)

☐ 2 Type 2 (Non-insulin dependent or Mature onset)

☐ 3 Don't know

☐ 4 Other (please specify) _______________

F5 **Other than gestational diabetes, when were you first told you had diabetes?**

(tick one box only)

☐ 1 Within the last twelve months

☐ 2 1 to 2 years ago

☐ 3 3 to 5 years ago

☐ 4 6 to 10 years ago

☐ 5 More than 10 years ago

☐ 6 Don't know

F6 **Have you ever been told by a doctor that your vision has been affected because of your diabetes?**

(tick one box only)

☐ 1 Yes

☐ 2 No

☐ 3 Unsure/Don't know

F7 **Have you ever had laser therapy on your eyes because of your diabetes?**

(tick one box only)

☐ 1 Yes

☐ 2 No

F8 **Have you ever had cataract surgery?**

(tick one box only)

☐ 1 Yes

☐ 2 No

F9 **Do you often suffer tingling, pins and needles, burning or pain, or loss of sensation in your feet, toes or lower limbs?**

(tick one box only)

☐ 1 Yes

☐ 2 No

G. RESPIRATORY

ASTHMA
Symptoms of asthma include cough, wheezing, shortness of breath and chest tightness when you don't have cold or respiratory infection.

G1 **During the past 12 months, did you have any symptoms of asthma?**

(tick one box only)
- ☐ 1 Yes
- ☐ 2 No
- ☐ 3 Don't know

G2 **During the past 12 months, did you take asthma medication that was prescribed or given to you by a doctor? This includes using an inhaler, puffer or nebuliser.**

(tick one box only)
- ☐ 1 Yes
- ☐ 2 No
- ☐ 3 Don't know

G3 **Have you ever been told by a doctor that you have asthma?**

(tick one box only)
- ☐ 1 Yes
- ☐ 2 No **(Go to G16)** →
- ☐ 3 Don't know **(Go to G16)** →

G4 **Do you still have asthma?**

(tick one box only)
- ☐ 1 Yes
- ☐ 2 No **(Go to G16)** →
- ☐ 3 Don't know **(Go to G16)** →

G5 **When were you first told you had asthma?**

(tick one box only)
- ☐ 1 Within the last twelve months
- ☐ 2 1 to 2 years ago
- ☐ 3 3 to 5 years ago
- ☐ 4 6 to 10 years ago
- ☐ 5 More than 10 years ago
- ☐ 6 Don't know

G6 **How do you rate your asthma severity?**

(tick one box only)
- ☐ 1 Not a problem
- ☐ 2 Mild
- ☐ 3 Moderate
- ☐ 4 Severe

G7 **In the last 12 months, how many times have you had an urgent visit to a general practitioner for asthma?**

1 Enter number of TIMES ___________
- ☐ 99 Don't know

G8 **In the last 12 months, have you had any hospital admissions for asthma where you stayed for at least one night in hospital?**

(tick one box only)
- ☐ 1 Yes
- ☐ 2 No **(Go to G10)** →
- ☐ 3 Don't know **(Go to G10)** →

G9 **How many days would you estimate?**

1 Enter number of days ___________
- ☐ 99 Don't know

G10 **In the last 12 months, have you had any days lost from work, school, home duties or usual activities from asthma?**

(tick one box only)

- ☐ 1 Yes
- ☐ 2 No
- ☐ 3 Don't know

G11 **How many days?**

1 Enter number of days __________

- ☐ 99 Don't know

G12 **How much do you feel your asthma limits what you do in each of the following areas?**

(tick ONE box in each row)

	A lot	*Some*	*Only a little*	*Not at all*	*Not applicable*
**Sport / Recreation**	☐	☐	☐	☐	☐
**Normal physical activities (work or home)**	☐	☐	☐	☐	☐
**Housekeeping**	☐	☐	☐	☐	☐
**Social activities**	☐	☐	☐	☐	☐
**Other physical activities**	☐	☐	☐	☐	☐

G13 **How often do you awaken during the night with asthma?**

(tick one box only)

- ☐ 1 Nightly
- ☐ 2 Most nights
- ☐ 3 About twice a week
- ☐ 4 Weekly
- ☐ 5 Monthly
- ☐ 6 Less often than monthly
- ☐ 7 Only at certain times of the year
- ☐ 8 Never

G14 **Do you have an asthma action plan?** (written instructions of what to do if your asthma is out of control)

(tick one box only)

- ☐ 1 Yes
- ☐ 2 No
- ☐ 3 Don't know

G15 **How often would you use a reliever medication?** (such as Ventolin, Airomir, Bricanyl, Combivent or Epaq)

(tick one box only)

- ☐ 1 More than 4 times a day
- ☐ 2 Daily
- ☐ 3 Most days
- ☐ 4 About twice a week
- ☐ 5 Weekly
- ☐ 5 Monthly
- ☐ 6 Less often than monthly
- ☐ 7 Only at certain times of the year
- ☐ 8 Never

BRONCHITIS

G16 **Have you ever been told by a doctor that you have CHRONIC bronchitis?**

(that is, episodes of coughing and or sputum/phlegm for 3 or more consecutive months, over 2 or more consecutive years)

(tick one box only)

- ☐ 1 Yes
- ☐ 2 No **(Go to G19)**
- ☐ 3 Don't know **(Go to G19)**

G17 **When were you first told you had CHRONIC bronchitis?**

(tick one box only)

- ☐ 1 2 years ago
- ☐ 2 3 to 5 years ago
- ☐ 3 6 to 10 years ago
- ☐ 4 More than 10 years ago
- ☐ 5 Don't know

G18 **Thinking about the last time you had CHRONIC bronchitis, were you prescribed antibiotics by your doctor?**

(tick one box only)

- ☐ 1 Yes
- ☐ 2 No
- ☐ 3 Don't know

EMPHYSEMA

G19 **Have you ever had emphysema?**

(tick one box only)

- ☐ 1 Yes
- ☐ 2 No **(Go to H1)**
- ☐ 3 Don't know **(Go to H1)**

G20 **Was your emphysema ever confirmed by a doctor?**

(tick one box only)

- ☐ 1 Yes
- ☐ 2 No **(Go to H1)**
- ☐ 3 Don't know **(Go to H1)**

G21 **When were you first told you had emphysema?**

(tick one box only)

- ☐ 1 Within the last twelve months
- ☐ 2 1 to 2 years ago
- ☐ 3 3 to 5 years ago
- ☐ 4 6 to 10 years ago
- ☐ 5 More than 10 years ago
- ☐ 6 Don't know

H. LUNG FUNCTION

The next series of questions relate to your lung function over the past three months.

H1 **In the past three months, during a typical day, have any of these made you short of breath?**

(tick one box only)

- ☐ 1 No activity: such as at rest, while sitting or lying down
- ☐ 2 Light activity: such as walking on level ground, shopping, washing or standing
- ☐ 3 Moderate activity: such as walking up a gradual hill, climbing less than three flights of stairs or carrying a light load on level ground
- ☐ 4 Vigorous activities: such as running, walking up a steep hill, climbing three or more flights of stairs or carrying a moderate load on level ground
- ☐ 5 Other (please specify) ________________
- ☐ 6 None **(Go to H3)**

H2 **In the past three months, how often were you short of breath?**

(tick one box only)

- ☐ 1 Occasionally
- ☐ 2 Most days
- ☐ 3 All of the time

H3 **In the past three months, did any of these make you wheeze?**

(tick one box only)

- ☐ 1 No activity: such as at rest, while sitting or lying down
- ☐ 2 Light activity: such as walking on level ground, shopping, washing or standing
- ☐ 3 Moderate activity: such as walking up a gradual hill, climbing less than three flights of stairs or carrying a light load on level ground
- ☐ 4 Vigorous activities: such as running, walking up a steep hill, climbing three or more flights of stairs or carrying a moderate load on level ground
- ☐ 5 Other (please specify) ________________
- ☐ 6 None **(Go to H5)**

H4 **In the past three months, how often did you wheeze?**

(tick one box only)

- ☐ 1 Occasionally
- ☐ 2 Most days
- ☐ 3 All of the time

H5 **In the <u>past three months</u>, how often did you cough?**

(tick one box only)

- ☐ 1 Never **(Go to H7)**
- ☐ 2 Occasionally or only during a cold or flu
- ☐ 2 Most days
- ☐ 3 Every day

H6 **In the <u>past three months,</u> when you coughed, how much sputum or phlegm did you produce?**

(tick one box only)

- ☐ 1 None
- ☐ 2 Just a little
- ☐ 3 Several tablespoons a day
- ☐ 4 A coffee cup or more a day

H7 **In the <u>past 12 months</u>, have you had any attacks of breathlessness?**

(tick one box only)

- ☐ 1 Yes
- ☐ 2 No **(Go to I1)**
- ☐ 3 Don't know **(Go to I1)**

H8 **In the <u>past 12 months</u>, have you had episodes of breathlessness at night or in the early morning?**

(tick one box only)

- ☐ 1 Yes
- ☐ 2 No
- ☐ 3 Don't know

H9 **In the <u>past 12 months</u>, have you been becoming more breathless and less able to do activities?**

(tick one box only)

- ☐ 1 Yes
- ☐ 2 No
- ☐ 3 Don't know

I. ALCOHOL

I1 **How often do you usually drink alcohol?**

(tick one box only)

- ☐ 1 I don't drink alcohol **(Go to J1)** →
- ☐ 2 Less than once a week
- ☐ 3 On 1 or 2 days a week
- ☐ 4 On 3 or 4 days a week
- ☐ 5 On 5 or 6 days a week
- ☐ 6 Every day

I2 **A Standard Drink is equivalent to a schooner of full strength beer, a glass of wine or a nip of spirits. On a day when you drink alcohol, how many drinks do you usually have?**

(tick one box only)

- ☐ 1 1 or 2 drinks
- ☐ 2 3 or 4 drinks
- ☐ 3 5 or 8 drinks
- ☐ 4 9 or 12 drinks
- ☐ 5 13 or 20 drinks
- ☐ 6 More than 20 drinks

J. SMOKING

J1 **Do you currently smoke?**

(tick one box only)

- ☐ 1 Yes
- ☐ 2 No **(Go to J3)**
- ☐ 3 Occasionally

J2 **How many cigarettes do you usually smoke a day?**

- 1 Enter number of cigarettes _________ **(Go to J6)**
- ☐ 2 Less than one **(Go to J6)**
- ☐ 3 Only smoke cigars or pipes **(Go to J6)**

`

J3 **Have you ever smoked regularly (that is, at least once a day)?**

(tick one box only)

- ☐ 1 Yes
- ☐ 2 No **(Go to K1***)*

J4 **How many cigarettes did you usually smoke a day?**

- 1 Enter number of cigarettes _____
- ☐ 2 Less than one
- ☐ 3 Only smoke cigars or pipes

`

J5 **How old were you when you last <u>gave up</u> smoking?**

- 1 Enter age ___________
- ☐ 2 Can't remember

J6 **At what age did you first <u>start</u> smoking daily?**

- 1 Enter age ___________
- ☐ 2 Can't remember

K. MENTAL HEALTH AND WELLBEING (GHQ12)

K1 **Have you recently … been able to concentrate on whatever you're doing?**

(tick one box only)

- ☐ 1 Better than usual
- ☐ 2 Same as usual
- ☐ 3 Less than usual
- ☐ 4 Much less than usual

K2 **Have you recently … lost much sleep over worry?**

(tick one box only)

- ☐ 1 Not at all
- ☐ 2 No more than usual
- ☐ 3 Rather more than usual
- ☐ 4 Much more than usual

K3 **Have you recently … felt that you are playing a useful part in things?**

(tick one box only)

☐ 1 More so than usual
☐ 2 Same as usual
☐ 3 Less useful than usual
☐ 4 Much less than usual

K4 **Have you recently … felt capable of making decisions about things?**

(tick one box only)

☐ 1 More so than usual
☐ 2 Same as usual
☐ 3 Less useful than usual
☐ 4 Much less than usual

K5 **Have you recently … felt constantly under strain?**

(tick one box only)

☐ 1 Not at all
☐ 2 No more than usual
☐ 3 Rather more than usual
☐ 4 Much more than usual

K6 **Have you recently … felt that you couldn't overcome your difficulties?**

(tick one box only)

☐ 1 Not at all
☐ 2 No more than usual
☐ 3 Rather more than usual
☐ 4 Much more than usual

K7 **Have you recently … been able to enjoy your normal day-to-day activities?**

(tick one box only)

☐ 1 More so than usual
☐ 2 Same as usual
☐ 3 Less useful than usual
☐ 4 Much less than usual

K8 **Have you recently … been able to face up to your problems?**

(tick one box only)

☐ 1 More so than usual
☐ 2 Same as usual
☐ 3 Less useful than usual
☐ 4 Much less than usual

K9 **Have you recently_… been feeling unhappy and depressed?**

(tick one box only)

☐ 1 Not at all
☐ 2 No more than usual
☐ 3 Rather more than usual
☐ 4 Much more than usual

K10 **Have you recently … been losing confidence in yourself?**

(tick one box only)

☐ 1 Not at all
☐ 2 No more than usual
☐ 3 Rather more than usual
☐ 4 Much more than usual

K11 **Have you recently … been thinking of yourself as a worthless person?**

(tick one box only)
- ☐ 1 Not at all
- ☐ 2 No more than usual
- ☐ 3 Rather more than usual
- ☐ 4 Much more than usual

K12 **Have you recently … been feeling reasonably happy, all things considered?**

(tick one box only)
- ☐ 1 More so than usual
- ☐ 2 Same as usual
- ☐ 3 Less useful than usual
- ☐ 4 Much less than usual

K13 **Are you currently taking any medications for mental health problems?**

(tick one box only)
- ☐ 1 Yes
- ☐ 2 No
- ☐ 3 Don't know

L. DEMOGRAPHICS

L1 **How would you best describe your family structure?**

(tick one box only)
- ☐ 1 A family with a child or children living with both biological or adoptive parents
- ☐ 2 A step or blended family
- ☐ 3 A sole parent family
- ☐ 4 Shared care parenting
- ☐ 5 Adult living alone
- ☐ 6 Adult living with partner and no children
- ☐ 7 Related adults living together
- ☐ 8 Unrelated adults living together
- ☐ 9 Other (specify) ________

L2 **What is your highest educational qualification?**

(tick one box only)
- ☐ 1 Still at school
- ☐ 2 Left school at 15 years or less
- ☐ 3 Left school after age 15
- ☐ 4 Trade/Apprenticeship
- ☐ 5 Certificate/Diploma
- ☐ 6 Bachelor degree or higher
- ☐ 7 Other (please specify) __________
- ☐ 8 Don't know

L3	**We are interested in how income relates to health, lifestyle and access to health services. Before tax is taken out, what was the amount of your household's income, from all sources, for the last 12 months?**	*(tick one box only)*<br>☐ 1 Up to $12,000<br>☐ 2 $12,001 - $20,000<br>☐ 3 $20,001 - $30,000<br>☐ 4 $30,001 - $40,000<br>☐ 5 $40,001 - $50,000<br>☐ 6 $50,001 - $60,000<br>☐ 7 $60,001 - $80,000<br>☐ 8 $80,001 - $100,000<br>☐ 9 More than $100,000
L4	**What is your marital status?**	*(tick one box only)*<br>☐ 1 Married or living with a partner<br>☐ 2 Separated / Divorced<br>☐ 3 Widowed<br>☐ 4 Never married
L5	**What is your work status?**	*(tick one box only)*<br>☐ 1 Full time employed<br>☐ 2 Part time / casual employment<br>☐ 3 Unemployed<br>☐ 4 Home duties<br>☐ 5 Retired<br>☐ 6 Student<br>☐ 7 Other *(please specify)* __________
L6	**Do you receive a pension or benefit from the Department of Social Security?** *(This does not include family allowance)*	*(tick one box only)*<br>☐ 1 Yes<br>☐ 2 No<br>☐ 3 Don't know
L7	**How old are you?**	*Enter age (years)* __________
L8	**What is your postcode?**	*Enter postcode* __ __ __ __

Thank you very much for taking the time to complete this questionnaire.

Please make sure that you have answered all the questions.
Please bring this questionnaire with you to the clinic appointment.

If you have any problems or questions in completing this questionnaire, please telephone Sandy (Clinic Co-ordinator) on ☏ 8222 7866 or Janet (Study Co-ordinator) on ☏ 8226 6054.
